# Spatial and temporal inequalities in mortality in the USA, 1968–2016

**DOI:** 10.1016/j.healthplace.2021.102586

**Published:** 2021-05-16

**Authors:** Welcome Wami, David Walsh, Benjamin D. Hennig, Gerry McCartney, Danny Dorling, Sandro Galea, Laura Sampson, Ruth Dundas

**Affiliations:** a MRC/CSO Social and Public Health Sciences Unit, Institute of Health and Wellbeing, University of Glasgow, Berkeley Square, 99 Berkeley Street, Glasgow, G3 7HR, Scotland, United Kingdom; b Glasgow Centre for Population Health, Olympia Building, 2-16 Orr Street, Bridgeton Cross, Glasgow G40 2QH, Scotland, United Kingdom; c Faculty of Life and Environmental Sciences, University of Iceland, Askja, Sturlugata 7, 101 Reykjavík, Iceland; d Adam Smith Business School, University of Glasgow, Glasgow G12 8QQ, Scotland, United Kingdom; e School of Geography & the Environment, University of Oxford, South Parks Road, Oxford OX1 3QY, England, United Kingdom; f School of Public Health, Boston University, 715 Albany Street - Talbot 301, Boston, MA 02118, USA; g Amsterdam Institute for Global Health and Development, AHTC, Tower C4, Paasheuvelweg 25, 1105 BP Amsterdam, Netherlands; h Harvard T.H. Chan School of Public Health, 677 Huntington Ave, Boston, MA 02118, USA

**Keywords:** ‘Excess mortality’, Poverty, Deprivation, USA

## Abstract

Previous UK and European research has highlighted important variations in mortality between populations after adjustment for key determinants such as poverty and deprivation. The aim here was to establish whether similar populations could be identified in the US, and to examine changes over time. We employed Poisson regression models to compare county-level mortality with national rates between 1968 and 2016, adjusting for poverty, education, race (a proxy for exposure to racism), population change and deindustrialisation. Results are presented by means of population-weighted cartograms, and highlight widening spatial inequalities in mortality over time, including an urban to rural, and south-westward, shift in areas with the highest levels of such unexplained ‘excess’ mortality. There is a need to understand the causes of the excess in affected communities, given that it persists after adjustment for such a broad range of important health determinants.

## 1 Introduction

Poverty, and broader socioeconomic deprivation, are amongst the most important determinants of health and mortality within and between populations (Galea et al., 2011). Related factors such as deindustrialisation (associated, for example, with unemployment, poverty and deskilling) and racial discrimination are also hugely important (Walsh et al., 2010; Rodriguez et al., 2015). In the US, for example, higher mortality rates have been noted for those living in poverty (Krieger et al., 2008) and, particularly, for Black Americans (Geronimus et al., 1999), leading to marked geographical, racial, and socioeconomic inequalities in health (Krieger et al., 2008; Satcher et al., 2005; Shiels et al., 2017; Bor et al., 2017; Wang et al., 2013). In the 1980s life expectancy fell notably among the Black population (Centers for Disease Control and Prevention, 2018), and since 1990, mortality improvements have stalled for the least well educated and poorest groups (Bor et al., 2017; Bound et al., 2015; Case and Deaton, 2015; Verguet and Jamison, 2013; Minton et al., 2017), particularly amongst Blacks and Whites in areas with high ethnic diversity (Deaton and Lubotsky, 2003). Across the country it is known that area poverty levels explain much of the variation in mortality rates between counties (Dwyer-Lindgren et al., 2017). Higher mortality has become concentrated in some poorer geographical areas around the Appalachian mountains and in the deep south (Wang et al., 2013; Kulkarni et al., 2011; Cullen et al., 2012), as well as within some inner city parts of deindustrialised urban areas (Murray et al., 2006). Marked deindustrialisation and economic distress in some US cities has been shown to be associated with worse health outcomes (Silver et al., 2011).

Similar socioeconomic drivers of health inequalities have also been shown in Europe (Mackenbach et al., 2008; Taulbut et al., 2014), including in the UK where marked socioeconomic and spatial inequalities in mortality rates have been observed, with substantially higher mortality amongst those who are working class (White and Edgar, 2010), live in the most deprived areas (McCartney et al., 2017), and who live in northern England and Scotland (Mitchell et al., 2000). The differences in health outcomes by ethnicity are more mixed but include, for example, higher mortality amongst those with a South-East Asian heritage living in England (Tunstall et al., 2007; Rees et al., 2009).

However, a body of research has also demonstrated that notable variations in mortality have been observed between populations even when differences in socio-economic disadvantage – *and, importantly, other health determinants such as medical care* – have been taken into account (Geronimus et al., 1999; Walsh et al., 2017; Walsh et al., 2016). For example, within the UK, studies have shown that the extent to which the high mortality observed in Scotland (and especially in its largest city, Glasgow) is attributable to poverty, deprivation and socioeconomic position has changed markedly over time (Schofield et al., 2016). Similarly, the mortality experiences of the most deindustrialised European regions have varied considerably, with less rapid improvements in countries with a more market-orientated approach to the economy (such as the UK) (Walsh et al., 2010; Taulbut et al., 2014; Taulbut et al., 2013). These studies suggest an important role of policy, and in particular neoliberalism, in the formulation of this type of unexplained excess mortality (Collins and McCartney, 2011; Daniels, 2014). Indeed, in Scotland’s and Glasgow’s case, a substantial body of recent research concluded that neoliberal politics combined with vulnerabilities resulting from historical urban and regional policies best explain why the populations have experienced these particular, adverse, mortality patterns (Walsh et al., 2016, 2017).

The role of policy has also been highlighted in an American context – for example, in relation to evidence suggesting that some populations are particularly vulnerable to changes in economic policy and experience worse health as a result (Galea et al., 2005), and in terms of the lack of a national urban planning system (as exists elsewhere) which has resulted in extremes of ‘ghost towns’ and vast urban sprawls unimpeded by ‘green belts’; the latter has been highlighted as a potential driver of further geographical inequalities by means of associated selective migration (Pattison, 2004; Uberti, 2014). Given the USA’s very similar political and policy trajectory to the UK’s over the last 40 years (Montez et al., 2020), it might be expected that the US would have similarly experienced increased levels of this form of unexplained ‘excess mortality’ (i.e. mortality over and above that explained by socioeconomic differences within and between nations, and influenced by particular forms of national and/or local policy-making). Although there are a number of similarities in health trends between the UK and the US – not least, the scale of inequalities, as well as the recent stalling of improvement in life expectancy (Fenton et al., 2019) – the extent to which this is the case has not been fully tested. Given all the above, the overall aim of this study was to identify whether or not there are geographically defined populations within the US which experience this type of unexplained excess mortality, as has been observed in the UK. The specific research questions were:

1. To what extent are differences in all-cause mortality rates across counties of the US explained by differences in area-level poverty and education?
2. How does this differ by time period, sex and age, and in relation to different causes of death?
3. To what extent do areas with unexplained excess mortality cluster within US states and larger regions?
4. To what extent are levels of excess mortality attenuated by adjusting for: race, county population change; deindustrialisation; or other state-level influences?

It is important to emphasise that in seeking to identify areas with such unexplained ‘excess’ mortality – i.e. adjusted for socioeconomic risk factors – the intention is **not** to overlook or understate the importance of those important socioeconomic determinants themselves: these are, after all, the ‘fundamental causes’ of health inequalities (Link and Phelan, 2002). Rather, it is to provide a starting point for a better understanding of any *additional* – e.g. political, historical – potential influences, *over and above* those well understood fundamental determinants.

## 2 Methods

### 2.1 Geography

US county (as defined in 1968) was the main spatial unit of analysis. Counties whose Federal Information Processing System (FIPS) codes had changed over time were back-mapped to codes used in 1968. In addition, following the methodology of Dwyer-Lindgren et al. (Dwyer-- Lindgrenet al, 2016), some small counties were merged to ensure stable units of analysis over time.

### 2.2 Time periods

Analyses were undertaken for the following time periods: 1968–78; 1979–88; 1989–98; 1999–2008, 2009–16. These periods were chosen to ensure that there was a sufficient number of deaths in each time period to facilitate the calculation of robust and reliable mortality rates at county level. In addition, the periods are aligned with changes to International Classification of Diseases (ICD) coding systems (ICD8, ICD9, ICD10).

### 2.3 Data sources

The data used in the analyses are described below, and the time periods covered by each data source are also summarised in online appendix Table S1.

Mortality data by county, age, sex, race, cause and year of death were obtained from the US Centers for Disease Control and Prevention (CDC) for the years 1968–1988 (available publicly from the CDC website/file server), and from the National Center for Health Statistics (NCHS) for the period 1989‖2016 (available only from within the US and subject to approval by the National Association of Public Health Statistics Information Systems (NAPHSIS)) (National Center for Health Statistics, 2007, 2017). Age at death was available for 13 bands (<1; 1–4; 5–9; 10–14; 15–19; 20–24; 25–34; 35–44; 45–54; 55–64; 65–74; 75–84; 85+ years). Race was recoded to White, Black and Other: a more detailed breakdown (e.g. Hispanic/non-Hispanic) was not available for the earlier years of data and thus the more limited set of categories was used for all time periods. Data were obtained for all-cause deaths and for ten specific causes: respiratory disease; ischaemic heart disease (IHD); cerebrovascular disease; all cancers; lung cancer; suicide; external causes; motor vehicle traffic accidents (MVTAs); alcohol related causes; drug related poisonings. ICD codes for each cause and time period are listed in online appendix Table S2. As stated in that Table, the definition of external causes overlaps with other causes of death (i.e. with MVTAs, intentional self-harm and drug-related poisonings).

Matching population denominator data for the same counties, age groups, race categories, time periods, and by sex, were accessed with the mortality data from the same data sources (i.e. CDC for 1968–1988, NCHS for 1989–2016), having been derived from US Census Bureau data. Population change (defined as the percentage change in the county population size between the beginning and end of each time period) was derived from the same data.

County poverty rates (defined as the percentage of the population with income below the poverty line) were downloaded from the US Census Bureau’s online ‘library’ for the census years of 1970, 1980, 1990, 2000, as well as estimates for 2010 based on 5-year data from the Bureau’s American Community Survey (ACS). Educational attainment levels (defined as the percentage of adults in each county aged 25+ years who graduated with at least a high school diploma) were also obtained from the U.S. Census Bureau’s 1970, 1980, 1990, 2000 Censuses, and – for 2012 – from the American Community Survey (based on a five-year average for 2012–2016) (as it was not included in the 2010 census).

Deindustrialisation rates (defined as the percentage change in the number of industrial jobs in each period) were calculated from Labor Market Information Bureau (ELMI) data. As these data are only available at state level, they were applied at that level to each county within each state. Industrial jobs were defined as manufacturing (North American Industry Classification System (NAICS) sectors 31–33), mining (NAICS sector 21), and utilities (NAICS sector 22).

### 2.4 Statistical analyses

First, directly age-standardised mortality rates per 100,000 population were calculated for each county, time period, for both sexes, for all ages and 0–64 years, using the 2000 U.S. standard population for all time periods.

For the main analyses all-cause mortality rates for each county were then compared with the US national mortality rate in each time period using multilevel Poisson regression models, adjusting for (1) age only and then (2) age, poverty and education. Separate models were run for each time period, sex, and age group. The heterogeneity of US counties in terms of their population size was taken account of in the multilevel models. Given the potential lag effects of socio-economic conditions on mortality outcomes, additional sensitivity analyses were undertaken to check if poverty/education levels in a previous ten-year period better explained variation in mortality than poverty/education in the same time period.

The data used in these analyses had a hierarchical structure, i.e. individual cells of death counts and population denominator by age categories were nested within counties. Thus, 2-level multilevel models were fitted. These included a county random effect (random intercept), with cell characteristics related to age group, county-level poverty and education rates specified in the fixed part of the model and the log of the population denominator for rates as an offset variable. Assuming the number of deaths (*λ_ij_* in the population followed a Poisson distribution, for the *i*-th age category (*i* = 1*,…,I*) of the *j*-th county (*j* ≠ 1,…,*J*), we used models which can be formulated as follows (in this example we adjust only for age, poverty and education):

$$\log\left(\lambda_{ij}\right)=\beta_{0}+\beta_{1}{\text{Age}}_{ij}+\beta_{2}{\text{Poverty}}_{j}+\beta_{3}{\text{Education}}_{j}+u_{0j}+\log\left(N_{ij}\right)$$

where *N_ij_* is the population at risk in the *i*-th age category of the *j*-th county (offset variable) and *u_oj_* is the county random effect (random intercept) and $u_{0j}∼N(0,\sigma_{u0}^{2}).$

The resultant predicted county random effects measured how much higher the predicted log-rates were in each individual county relative to the national US average mortality rate. The estimated county-level random effect (*u_oj_*) (Leyland and Groenewegen, 2020) were plotted against their rank order, with 95% CIs, to identify counties with ‘excess’ mortality (in comparison to the US average mortality rate). The residuals were further categorised as: >20% below average; 20-10% below average; average (10% below to 10% above); 10–20% excess; >20% excess. These were derived from the following residuals’ values (note that the values of the residuals correspond to log(0.8), log(0.9), log (1.1), log(1.2) which determined the percentage excess): < –0.22; –0.22 to –0.105; –0.105 to 0.095; 0.095 to 0.18; >0.18.

To explore further potential influences on excess mortality, three additional sets of multilevel Poisson models were fitted adjusting for race, population change and deindustrialisation (in addition to age, poverty and education). In addition, we examined interactions between poverty, education and race, but the models including these interactions did not explain greater variations in the outcome; hence by principle of parsimony, we adopted the simpler models. We also undertook preliminary descriptive examination of the relationship between poverty rates, education rates and mortality rates. There was no apparent nonlinear relationship that could be assumed and therefore we chose not to include these higher order terms in the models. All models were fitted in SAS 9.4 using the PROC GLIMMIX procedure.

To quantify the variation in excess mortality explained in the different models, we calculated the proportions of counties in each of the five excess groups (as described above) in each time period, and also plotted the ranked estimated residuals (caterpillar plots) with 95% confidence intervals.

The contribution of different causes of death across the five groups of counties (i.e. with different levels of excess mortality) were assessed by analysing age-standardised rates for each cause in each time period, including as a percentage of the all-cause death rate.

The potential influence of US states on county mortality rates was assessed, first by analysing the extent to which areas with excess were clustered within states and, second, by running extra models which included state as a third level (with counties nested within them).

Mortality rates and levels of residuals-defined excess mortality were examined not just in tabular and standard GIS mapping format, and also by means of population-weighted cartograms, the latter used to identify populations that can be ‘hidden’ within standard GIS-based spatial mapping. These ‘density-equalising’ maps were used to help identify both state and regional clusters of excess mortality. The maps are based on methods described elsewhere (Gastner and Newman, 2004) and which have been deployed within the scope of the ‘Worldmapper’ project at global, regional and local scale (Hennig, 2013; Hennig, 2019). The visualisations focus on the contiguous United States and therefore exclude Alaska and Hawaii – these would be difficult to see given their very small population size.

**Fig. 1:**
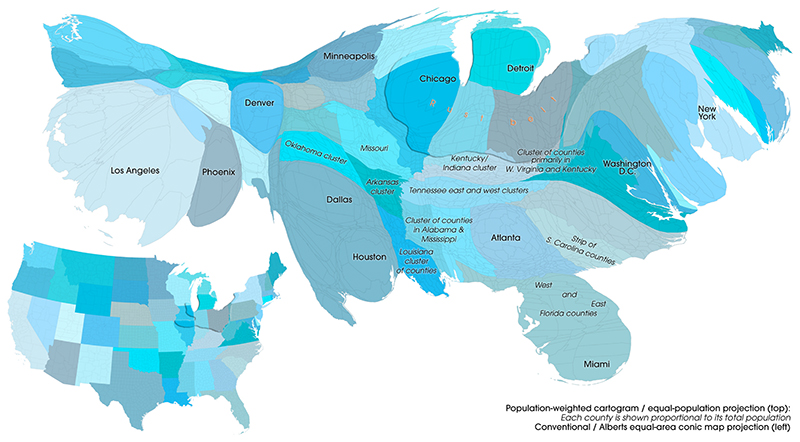
Reference map, showing the cartogram alongside a ‘normal’ map and including key geographic features for orientation: States are coloured differently; the rust belted is highlighted in both; clusters of particular counties discussed the paper are also highlighted.

A geographical guide to these cartograms is presented in Fig. 1. This compares a conventional map of the U.S. with an example cartogram. In the latter, the size of each county is proportional to its population size: thus, more heavily populated counties, including those containing all or part of cities, are larger. N.B: To assist clarity, Fig. 1 also identifies particular cities and clusters of counties which are referred to in the description of the results below.

In all analyses, counties with less than 10 deaths in each ten-year time period, and deaths for which age or county code was unknown, were excluded from the analyses.

## 3 Results

Table S3 in the online appendix summarises the distribution of age-standardised all-cause mortality rates across US counties in the five time periods analysed (1968–78, 1979–88, 1989–98, 1999–2008, 2009–16), and shows improving median rates over time for deaths when collapsed across all ages, but either a stalling of improvement (males) or a slight worsening (females) for premature deaths (0–64 years).

**Fig. 2:**
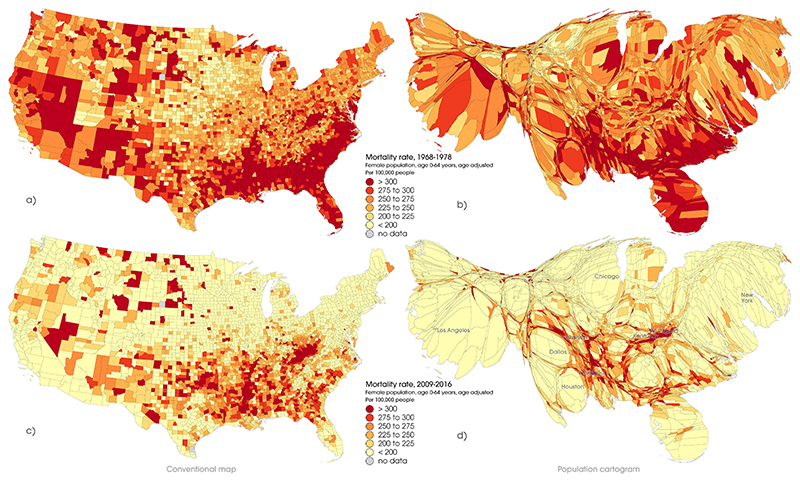
Age-standardised mortality rates by US county, females, 0–64 years, 1968–78 and 2009–16 presented in conventional maps (left) and population-weighted cartograms (right). Source: National Center for Health Statistics (Compressed Mortality Files, 1968–88, 1989–98, 1999–2016).

Female premature mortality rates for all counties are shown graphically in Fig. 2 for the first (1968–78) and last (2009–16) time periods. This includes both conventional spatially accurate maps and population-weighted cartograms described above.

Fig. 2 shows both the general reduction in rates of premature mortality between the two time periods and clusters of persistently higher mortality in (for example) West Virginia, Kentucky, Arkansas and Louisiana. The relative improvements in mortality in urban areas such as Chicago, Los Angeles, New York, Houston and Dallas are very marked. The same data for males 0–64 years are presented in online appendix Fig. S1, and for males and females of all ages in Figs. S2 and S3 respectively.

**Fig. 3:**
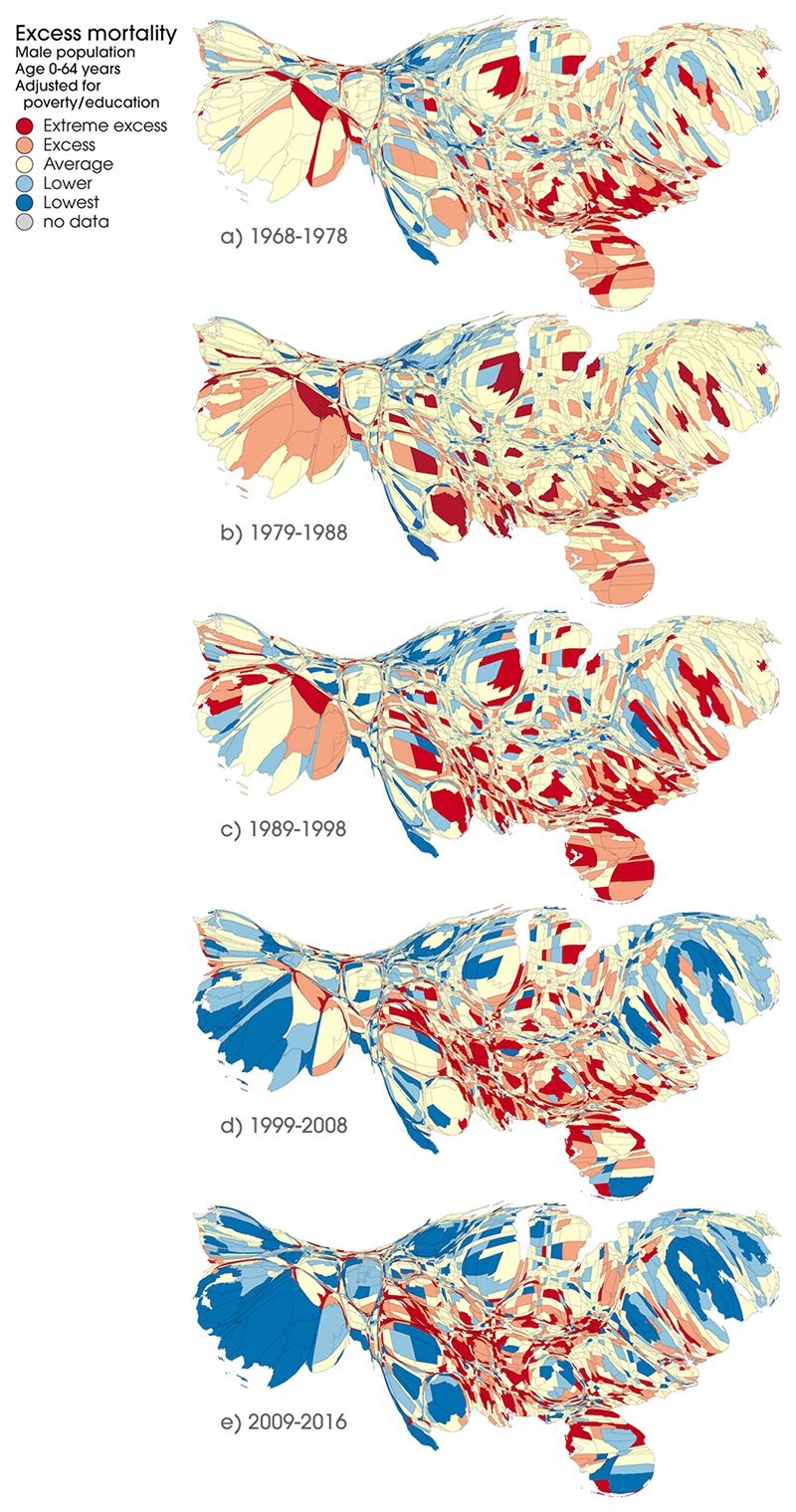
Excess mortality by US county, adjusted for age, poverty and education, males, 0–64 years, 1968–78 to 2009–16. Source: National Center for Health Statistics (Compressed Mortality Files, 1968–88, 1989–98, 1999–2016).

Fig. 3 presents the residuals-defined levels of excess mortality, after adjustment for poverty and education, for all US counties in each of the five time periods. These data are for males 0–64 years; equivalent data for females are presented in Fig. S4. The maps highlight a number of changing trends in poverty/education-adjusted mortality in the US.

First, in the earliest period, the highest levels of excess mortality can be seen in urban areas such as Chicago, Detroit, New York, Atlanta and around Las Vegas, as well as in large parts of North and South Carolina. However, by the latest period, the areas with the highest rates are instead clustered across smaller, less urban, parts of the southern states, e.g. within Louisiana, Alabama, Mississippi, Tennessee, Florida, as well as parts of West Virginia, Kentucky, Indiana, Missouri, Oklahoma and Texas. In contrast, many urban areas (e.g. Los Angeles, Dallas, Houston, Washington DC, New York, Chicago) have the lowest adjusted rates in the final period of analysis. This changing pattern also includes a noticeable westward shift in areas with the highest excess mortality.

A change in the spatial arrangement of mortality is particularly evident between 1989–98 and 1999–2008, with the concentration of clusters of excess mortality in the south and the emergence of lower levels of mortality in more populated urban areas: this striking patterning of spatial inequalities develops further in the final time period (2009–16).

It is also notable that despite persistent relatively high mortality in Detroit, the larger ‘rustbelt’ area that extends around that city (including Chicago, Baltimore, Pittsburgh and other post-industrial urban settlements) does not exhibit notably high levels of excess mortality compared with elsewhere in the country.

Equivalent data for all ages combined (not only 0–64 years), are shown in Figs. S5 and S6. These show broadly similar changing patterns, but with lower excess mortality compared with the premature deaths only.

The extent to which excess mortality appears concentrated around and within particular states is quantified in Tables S4–S7 in the online appendix. These tables show, for males and females (all ages and 0–64 years only), the distribution of counties with excess mortality across states in each of the five time periods. For example, for males aged 0–64 years, in the earliest period almost 14% (n = 89) of all counties with excess mortality were located within Georgia, with approximately one third (32%) of such counties located in Georgia, South and North Carolina, and Florida. However, in the final period, one third of all such counties were instead located within Texas (9%), Tennessee (7%), Oklahoma (7%), Kentucky (6%) and Georgia (5%), reflecting the partial westward shift visible in Fig. 3. However, unlike the cartograms, these tables do not take county population size into account.

Figs. S7–S10 show the relative contribution of the different causes of death in the five different time periods for the five groups of adjusted-mortality-defined counties (i.e., the counties with the lowest excess mortality through to highest levels of excess mortality). Few notable differences are evident across the groups. For premature deaths (Figs. S7–S8), the greatest contributions were from external causes and all cancers – this was true across all groups and all time periods. The relative contribution of ischaemic heart disease (IHD) and stroke declined over time, while the contributions of suicide and drug-related poisonings increased between the first and last period: however, these patterns were again generally similar across all five groups of counties. For alcohol-related causes, the biggest increases were between 1989–98 and the later periods (again, observed across all groups). All groups also saw an increasing contribution of external causes, although the increase was slightly less pronounced among counties with the lowest levels of poverty- and education-adjusted mortality. The contribution of motor vehicle transport accidents (MVTAs) decreased slightly among counties with the lowest adjusted mortality rates, and increased slightly among those with the highest levels of excess mortality. Broadly similar results were observed for deaths at all ages (S9–S10), although for all ages combined, the contribution of drug-related poisonings peaked in 1989–98, rather than in 2009–16 (as was the case for 0–64 years). Furthermore, the greatest contributions (across all time periods and groups of counties) were from cancer and IHD, with external causes much less relevant.

**Fig. 4:**
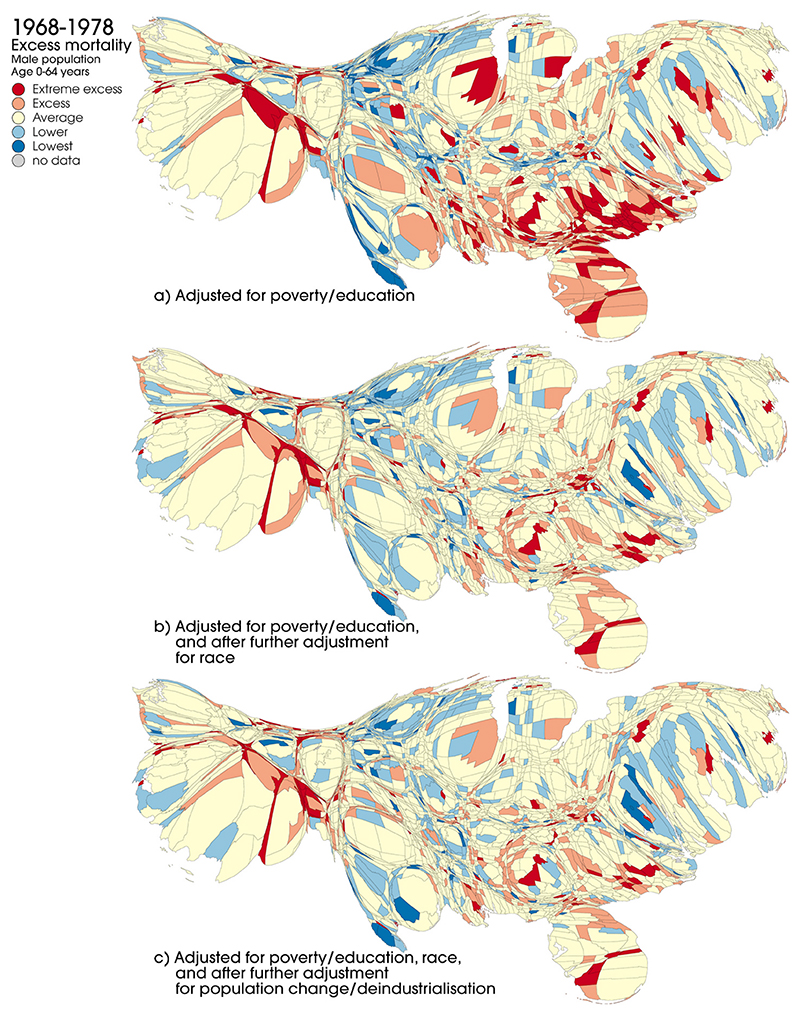
Excess mortality by US county, males, 0–64 years, adjusted for (a) age, poverty and education, (b) age, poverty, education and race and (c) age, poverty, education, race, population change and deindustrialisation, males, 0–64 years, 1968–78. Source: National Center for Health Statistics (Compressed Mortality Files, 1968–88, 1989–98, 1999–2016).

**Fig. 5:**
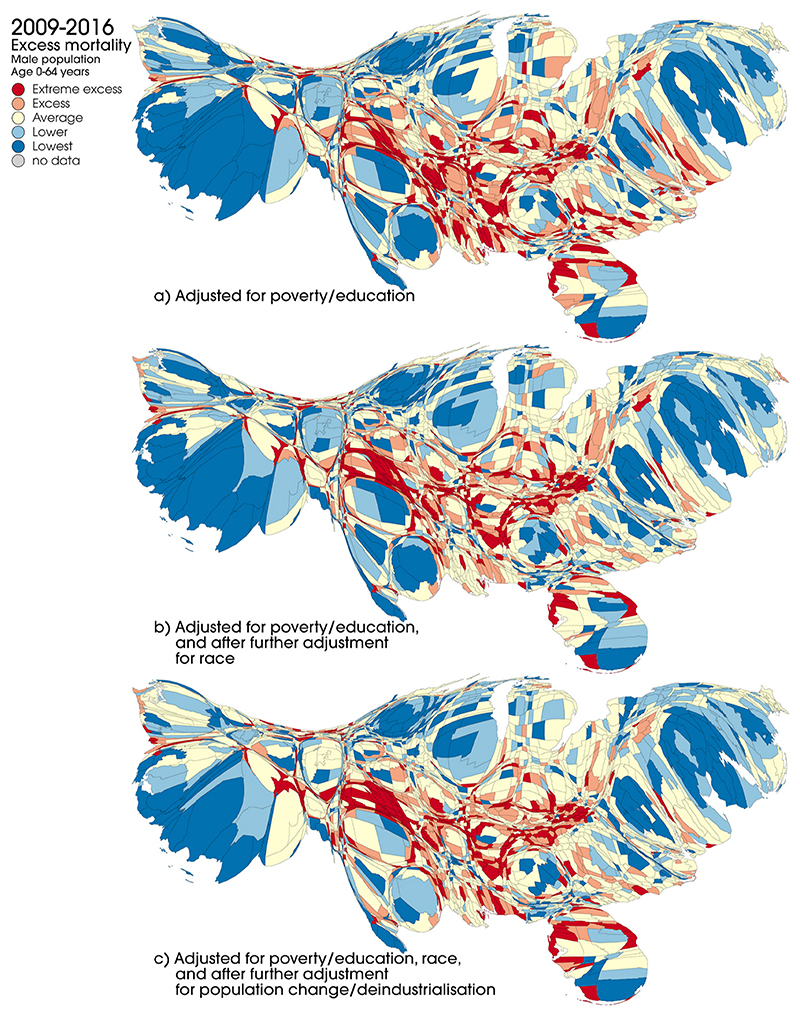
Excess mortality by US county, males, 0–64 years, adjusted for (a) age, poverty and education, (b) age, poverty, education and race and (c) age, poverty, education, race, population change and deindustrialisation, males, 0–64 years, 2009–16. Source: National Center for Health Statistics (Compressed Mortality Files, 1968–88, 1989–98, 1999–2016).

Figs. 3 and 4 compare county-level mortality rates after adjustment for poverty and education (as shown in Fig. 3) and after further adjustment for race, population change and deindustrialisation. These rates are shown for the first period (Fig. 4) and the last period (Fig. 5) for males aged 0–64 years. Fig. S11 show these data for all five periods together; Fig. S12 presents the data for females, and Figs. S13–S14 show the data for deaths at all ages. In the first period, adjustment for race appears to have a visible effect in reducing the number and concentration of counties with the highest levels of excess mortality; in the last period, however, the effect appears less pronounced. With some individual exceptions, further adjustment for population change and levels of deindustrialisation does not appear to notably alter the overall spatial patterning of excess mortality across the US. After adjustment for all factors, the areas with the highest levels of excess mortality by 2009–16 are still largely those shown in Fig. 3 (i.e. after adjustment for poverty and deprivation only): in particular clusters of counties within Florida, West Virginia, Kentucky, Tennessee, Alabama, Mississippi, and Oklahoma. Broadly similar patterns are observed for females (Fig. S12).

The extent to which adjustment for the other variables (race, deindustrialisation, population change) impacts on levels of excess mortality is quantified in Table S8, showing the proportions of counties in each excess mortality category and time period for each model. As alluded to above, this confirms that in the earlier periods adjustment for race has a greater impact than in the later periods: for example for males 0–64 years in the 1968–78 period, 21.9% of counties are classed as having excess or extreme excess mortality after adjustment of poverty and education alone (model 1), but this reduces to 17.7% after further adjustment for race (model 2). However, the equivalent figures for 2009–16 are 31.3% and 30.6% respectively. The Table also confirms that the impact of further adjustment for deindustrialisation and population change is fairly minimal in all periods. The increase in levels of excess mortality over time shown in Figs. 4 and 5 is also confirmed and quantified.

The final model, adjusting for any further state-level influences, explained little additional variation in outcomes (data not shown).

## 4 Discussion

We show an analytical and graphical depiction of changing levels and patterns of unexplained excess mortality in the US over almost half a century. As part of a picture of widening spatial inequalities, these findings highlight an urban to rural, and part westward, shift in such excess mortality with communities within a number of southern states particularly adversely affected. After adjustment for poverty, education, race, population change and levels of deindustrialisation, by 2009–16 large clusters of counties from Oklahoma and Texas through to Florida exhibit the highest levels of excess mortality (with a particular change evident from 1989/98 onwards). Adjustment for race makes a greater difference to the excess mortality in the earlier time periods than later.

Our findings correspond with those of a number of other studies. The widening of socio-economic and spatial inequalities in mortality in the US have been discussed by a number of authors (Case and Deaton, 2015; Fenelon and Boudreaux, 2019; Dobis et al., 2020), with the urban-rural shift an important component of those changes. For example, Cosby et al. showed a widening urban-rural mortality gap between the mid-1980s and 2016, highlighting the interaction of poverty and rurality as the most important determinants (alongside education and racism) (Cosby et al., 2019). In contrast, the authors pointed to faster mortality improvements in urban areas, which echo our own results, and which were examined in more detail recently by Fenelon & Boudreaux (Fenelon and Boudreaux, 2019). The latter study showed ‘remarkable increases’ in life expectancy in particular cities between 1990 and 2015, and the authors contrasted those trends with those of some rural counties where life expectancy has not changed since the mid-1980s; it also showed that life expectancy in a number of southern states was falling behind areas in the northeast and west coast, echoing our findings. Other authors have recently summarised some of these changing trends under the heading of ‘the southern rural health and mortality penalty’ (Miller and Vasan, 2021).

These widening spatial inequalities are clearly associated with some of the adverse mortality trends at the national level that have been much publicized of late. This includes evidence of worsening mortality among the middle-aged White population in the early years of the 21st Century, driven in particular by increases in suicide and alcohol- and drug-related deaths (Case and Deaton, 2015; Case and Deaton, 2017). Although these trends were contrasted with improving rates among middle-aged Black Americans in those years, more recent work (e.g. by Woolf et al. (2018)) has shown similarly worsening mortality among the middle-aged Black population since around 2011. Importantly, Muennig et al. have argued that these many different recent trends (by ethnicity, age, geography) are just the latest instalment in a much longer-term decline in health and longevity experienced in the US in comparison with other developed nations (Muennig et al., 2018). They argue this is driven by persistent structural problems affecting the whole population but disproportionately the poorest, and point to symptomatic trends in worsening rates of stress and depression, alongside a range of probable causes including democratic failure and insufficient social security systems, economic stagnation, income inequalities and a healthcare system characterised by spiralling costs. Many of these trends echo arguments of other authors (Case and Deaton, 2017; Hill and Jorgenson, 2018; Knapp et al., 2019).

This longer-term perspective is important, given the evidence of the causes of excess mortality in other countries. In Scotland, for example, waves of adverse political decision-making over a number of decades have been shown to be an important causal factor for the excess mortality through creating harmful levels of vulnerability within the population (Walsh et al., 2017; Walsh et al., 2016). This suggests common social and economic policy implications of that work and of this US study, despite the clearly profound differences in context, not least in relation to the different influences of racism and rurality in the US (rural areas tending to be on average poorer in the US, the opposite to the UK), and in terms of the experiences of the most deindustrialised regions in Scotland and the UK. Thus, the most obvious shared implication of thisand the previous Scottish work concerns the political economy. The impacts of political structures and decision-making (particularly economic decision-making) on population health and, especially, health inequalities are well understood (Navarro and Shi, 2001; McCartney et al., 2019; Rodriguez, 2018): for example, the widening of health inequalities within Scotland (and the rest of the UK) under right-wing Conservative UK governments is well-known (Thomas et al., 2010; Walsh et al., 2020), as are the different health outcomes associated with Republican and Democrat administrations in the US (Rodriguez, 2019). However, the interaction between the political economy, socioeconomic determinants of health and structural racism is clearly much more relevant in the US, given both its greater ethnic diversity, and its longer and much more complex history of such matters (Inwood, 2015; Ash and Robinson, 2009).

Other implications of this study include the need for further work to understand the causes (including potential local influences) of high levels of unexplained excess mortality in areas of the southern US, especially given the fact that the excess persists after adjustment for such important factors as poverty, education, ethnicity and more. This could build upon recent work identifying the important role of political decision-making and policy in explaining differing trends across the US (Montez et al., 2020). Also relevant to this is that adjustment for race appears to explain more of the variation in mortality in the earlier time periods compared to the later periods. This could indicate: some progress over time in reducing structural racism; greater racial diversity within counties such that it becomes a less useful discriminator of populations who experience racism; or increased correlation between race, education and poverty at country level over time. More detailed analyses of these correlations, and of the county-level relationship between unadjusted mortality rates, and rates adjusted by poverty, education, and race over different time periods might be beneficial in this respect. Finally, in highlighting the scale of existing health inequalities in the US, the work provides important context for understanding the levels of the more recent COVID-19 inequalities that have been recorded (Dorn et al., 2020).

There are some limitations of the study, primarily due to restrictions of data and variables available for analysis. For the last analysis period, the data on poverty and education covered slightly different years (although this is unlikely to impact on the analyses). We used relatively large time periods of approximately 10 years, limited race categories and only state-level deindustrialisation variables. Thus, the results demonstrate a broad overview of excess mortality in US counties across the 50-year period and are likely conservative estimates. In addition, US county is not an optimal spatial unit, given the heterogeneity of population size. Some counties are exceptionally large compared to others; however, the use of the density-equalising maps minimises risks of overinterpretation of results for counties with small populations. Finally, although the modelling adjusted for population change between periods, this is unlikely to capture potentially broader impacts of inward and outward migration. The evidence regarding this paints a highly complex picture of fluctuations in population growth and decline, both over time and across different geographical areas. Since the turn of this century, rural areas and small towns have generally experienced large increases in population sizes (reversing previous declines of the 20th century), driven in particular by increases in the size of the Hispanic population; however, this has not been ubiquitous, with notable decreases in population size observed in areas such as the South, Midwest and Appalachia, associated with declining farming and other industries (Miller and Vasan, 2021).

The major strengths of this study include identification of communities with high levels of unexplained excess mortality, and demonstrating the benefits of novel mapping techniques. Other strengths include the pan-contiguous county coverage, the broad set of factors adjusted for in the modelling which has previously not been done at county level across the US, and the fact that the modelling took county population size into account.

## Supplementary Material

Supplementary Material
