# Supplementary material for: Spatial and temporal inequalities in mortality in the USA, 1968–2016
Source: Health Place. Author manuscript; Available in PMC 2022 Sep 7. (PMC7613337; doi:10.1016/j.healthplace.2021.102586)
Supplement: Supplementary Material [file EMS152039-supplement-Supplementary_Material.pdf]

Table S1. Time periods relating to main data sets used in analyses

Analysis period	ICD Code	Deaths	Population denominator	Race	Poverty rates	Educational attainment	Deindustrialisation*	Population Change
1968-1978	ICD-8	1968-1978	1968-1978	1968-1978	1970	1970	1970-1980	1968-1978
1979-1988	ICD-9	1979-1988	1979-1988	1979-1988	1980	1980	1980-1990	1979-1988
1989-1998	ICD-9	1989-1998	1989-1998	1989-1998	1990	1990	1990-2000	1989-1998
1999-2008	ICD-10	1999-2008	1999-2008	1999-2008	2000	2000	2000-2010	1999-2008
2009-2016	ICD-10	2009-2016	2009-2016	2009-2016	2010	2012**	2010-2016	2009-2016

**Industrial job losses for the different time periods (change from start year to end year): for the 1970s (1970–1980), 1980s (1980–1990), 1990s (1990-2000), 2000s (2000–2010), 2010s (2010–2016).*

***From the American Community Survey 5-year average (2012-2016).*

Table S2. - ICD codes used in cause-specific mortality analyses

Cause	ICD8 codes	ICD9 codes	ICD10 codes
Respiratory disease	460–519	460–519	J00-J99
Ischaemic heart disease	410-414	410-414	I20-I25
Cerebrovascular disease	430-438	430-438	I60-I69
All malignant neoplasms	140-209	140-208	C00-C97
Lung cancer (malignant neoplasm of trachea/bronchus/lung)	162	162	C33-C34
Intentional self-harm (including events of undetermined intent)	E950-E959, E980-E989	E950-E959, E980-E989	X60-X84, Y10-Y34, Y87.0, Y87.2
External causes	E800-E999	E800-E999	ICD10 V01-Y98
Motor vehicle traffic accidents (MVTAs)	E810-E819	E810-E819	V02-V04, V09, V12-V14, V19-V79, V86-V89
Alcohol related causes	262,291,303, 571.0, 571.9, E860	291, 303, 305.0, 425.5, 571.0-571.5, 571.8, 571.9, E860	F10, G312, G621, I426, K292, K70, K73, K740-K742, K746-K749, K860, X45, X65, Y15
Drug related poisonings	304, E8530-E8539, E8540-E8549, E8550-E855.6, E8560-E8569, E950, E962, E980	304, 305.2-305.9, E850-E858, E950.0-E950.5, E9620, E980.0-E980.5	F11-F16, F18, F19, X40-X44, X60-X64, X85,Y10-Y14

Note that there are overlaps between a number of categories e.g.: all cancers and lung cancer; all external causes, intentional self-harm and drug-related poisonings; external causes and MVTAs

Table S3. Summary distribution of age-standardised mortality rates per 100,000 population at U.S. county-level by time period, 1968-2016.

		**1968-1978**	**1979-1988**	**1989-1998**	**1999-2008**	**2009-2016**
	**Ages**	**Median (IQR)***	**Median (IQR)***	**Median (IQR)***	**Median (IQR)***	**Median (IQR)***
**Males**	0-14 years	190 (161-229)	122 (102-146)	65 (52-82)	54 (42-71)	47 (35-65)
	0-64 years	526 (463-614)	412 (356-483)	350 (296-420)	322 (264-392)	320 (263-395)
	15-44 years	277 (225-344)	217 (175-269)	205 (164-259)	198 (156-252)	196 (156-247)
	45-64 years	1,331 (1,180-1,532)	1,074 (922-1,255)	904 (767-1088)	811 (663-987)	813 (654-1012)
	65+ years	8,166 (7,660-8,680)	7,326 (6,857-7,739)	6,846 (6,363-7,340)	6,066 (5,571-6,612)	5,329 (4,856-5,856)
	All ages	1,499 (1,396-1,610)	1,287 (1,194-1,386)	1,169 (1,073-1,286)	1,046 (942-1,169)	954 (853-1,080)
**Females**	0-14 years	143 (119-174)	90 (75-112)	49 (38-64)	42 (32-56)	37 (28-52)
	0-64 years	260 (230-301)	211 (186-243)	192 (166-227)	189 (156-230)	197 (156-248)
	15-44 years	124 (102-154)	94 (78-115)	92 (74-117)	102 (79-135)	109 (82-143)
	45-64 years	640 (555-731)	559 (487-634)	524 (454-606)	497 (408-592)	514 (409-640)
	65+ years	5,285 (4,925-5,634)	4,602 (4,303-4,877)	4,508 (4,195-4,781)	4,487 (4,105-4,838)	4,109 (3,746-4,498)
	All ages	901 (840-970)	770 (717-824)	741 (683-799)	734 (661-810)	693 (618-779)
**Both**	0-14 years	172 (147-204)	112 (95-134)	61 (50-80)	52 (41-69)	48 (37-68)
	0-64 years	392 (348-453)	313 (276-362)	274 (236-325)	260 (216-314)	265 (217-325)

	15-44 years	204 (168-252)	160 (131-196)	155 (125-191)	155 (124-197)	159 (126-201)
	45-64 years	973 (865-1,105)	807 (707-925)	712 (609-838)	651 (537-785)	661 (536-822)
	65+ years	6,480 (6,114-6,864)	5,656 (5,358-5,966)	5,402 (5,073-5,738)	5,129 (4,719-5,536)	4,640 (4,241-5,055)
	All ages	1,170 (1,096-1,249)	993 (925-1,060)	924 (854-1,002)	875 (791-969)	817 (734-919)

*IQR is the interquartile range, the points between which the middle 50% of the population sit.

Source: National Center for Health Statistics (Compressed Mortality Files 1968-88, 1989-98, 1999-2016)

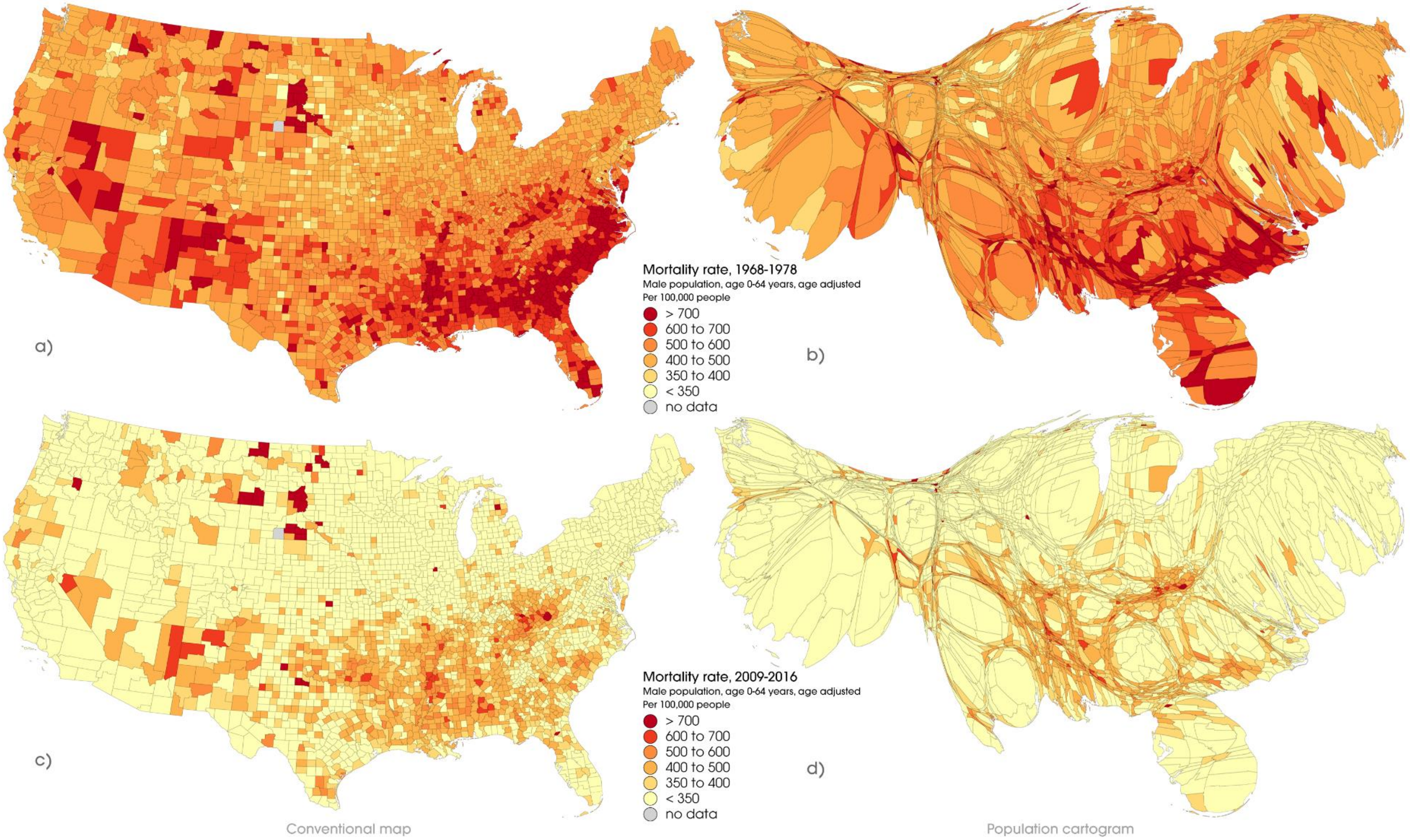

Fig. S1. Age-standardised mortality rates by US county, males, 0-64 years, 1968/78 and 2009/16 presented in conventional maps (left) and population-weighted cartograms (right). *Source: National Center for Health Statistics (Compressed Mortality Files 1968-88, 1989-98, 1999-2016)*

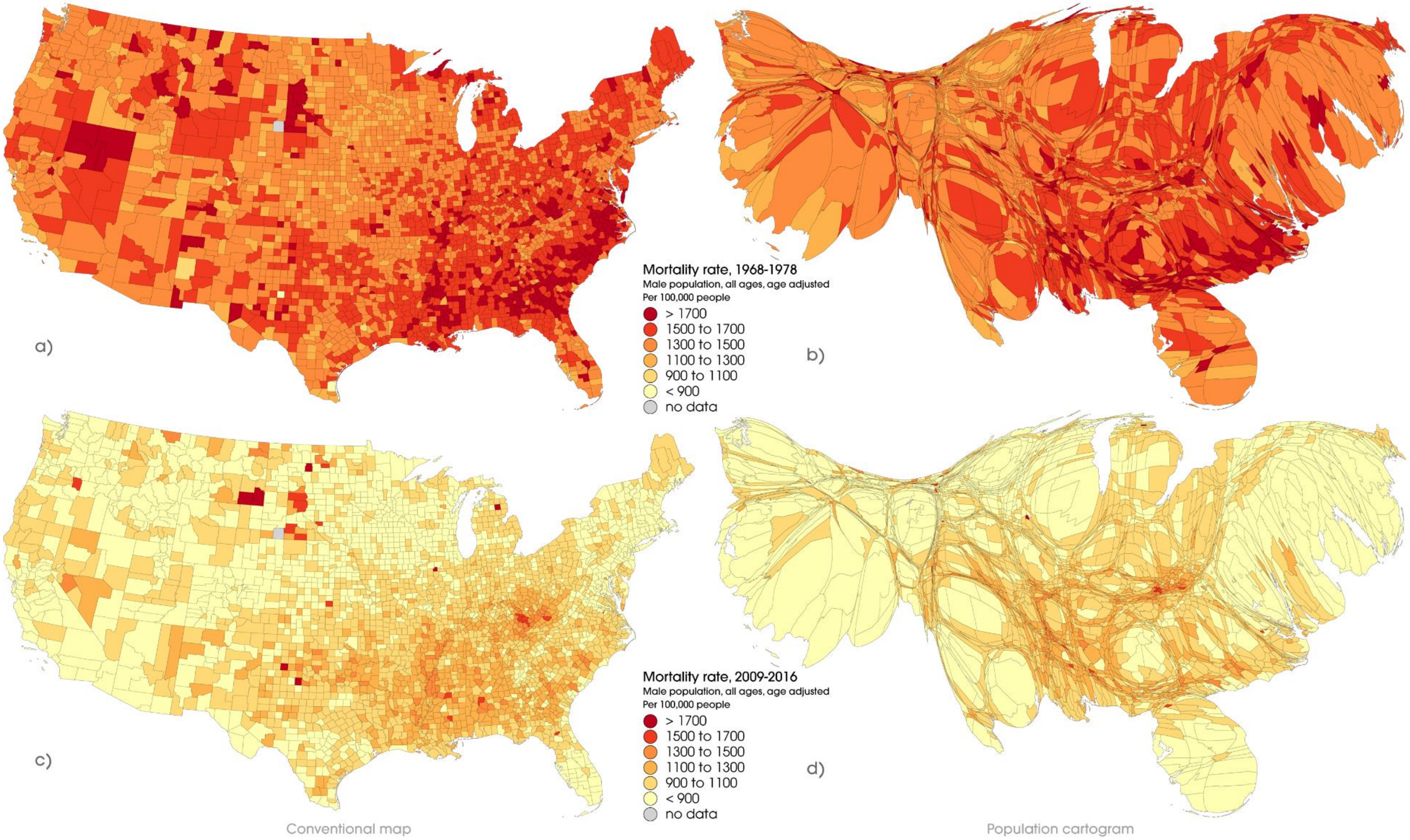

Fig. S2. Age-standardised mortality rates by US county, males, all ages, 1968/78 and 2009/16 presented in conventional maps (left) and population-weighted cartograms (right). *Source: National Center for Health Statistics (Compressed Mortality Files 1968-88, 1989-98, 1999-2016)*

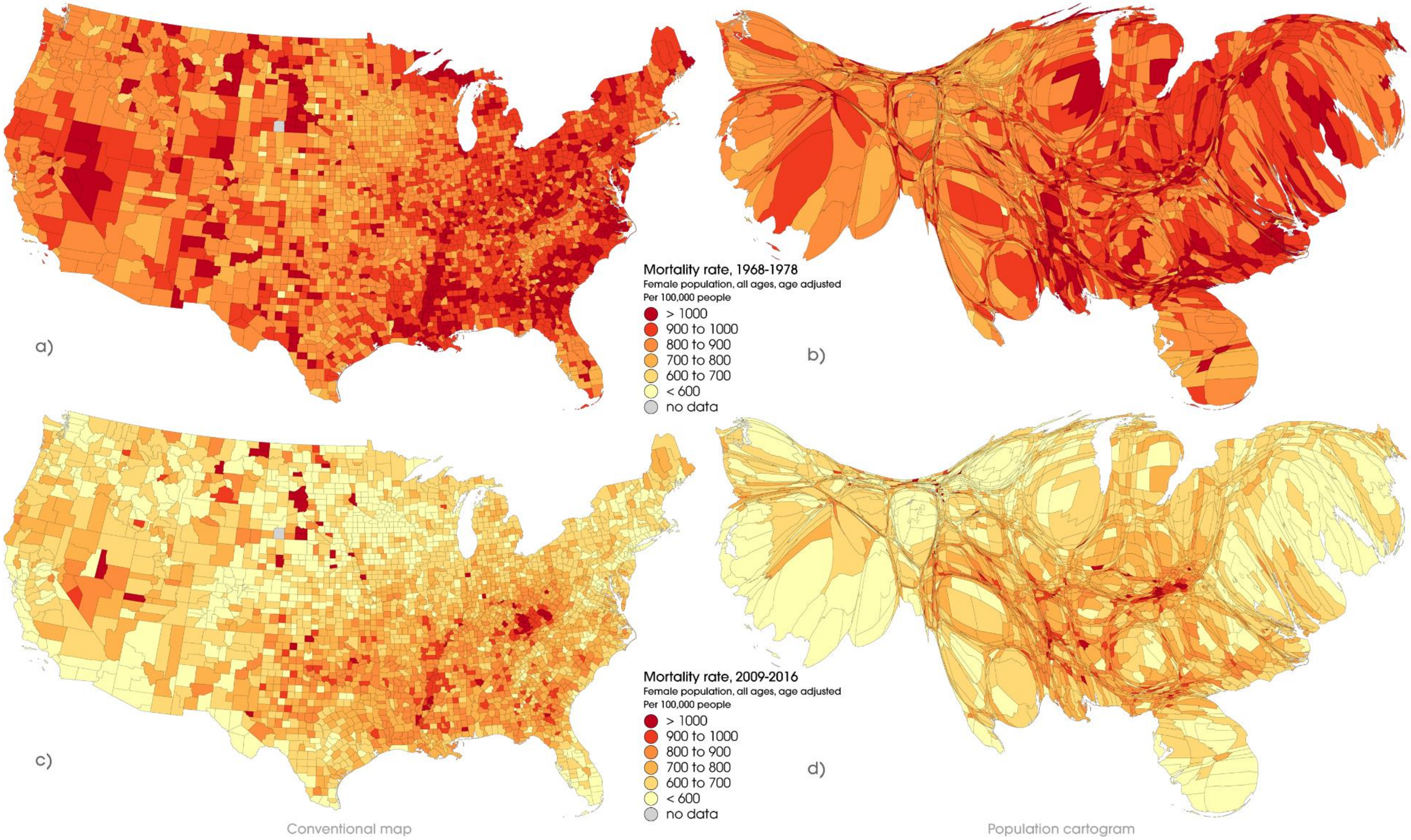

Fig. S3. Age-standardised mortality rates by US county, females, all ages, 1968/78 and 2009/16 presented in conventional maps (left) and population-weighted cartograms (right). *Source: National Center for Health Statistics (Compressed Mortality Files 1968-88, 1989-98, 1999-2016)*

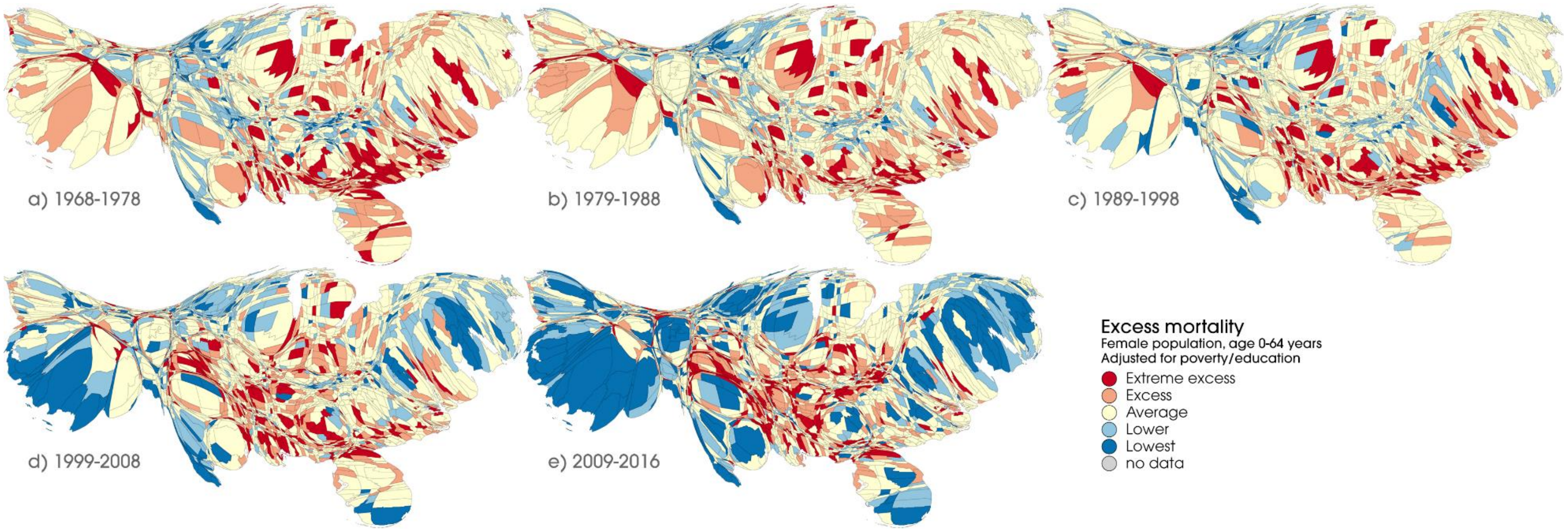

Fig. S4. Excess mortality by US county, adjusted for age, poverty and education, females, 0-64 years, 1968/78 to 2009/16.

Source: National Center for Health Statistics (Compressed Mortality Files 1968-88, 1989-98, 1999-2016)

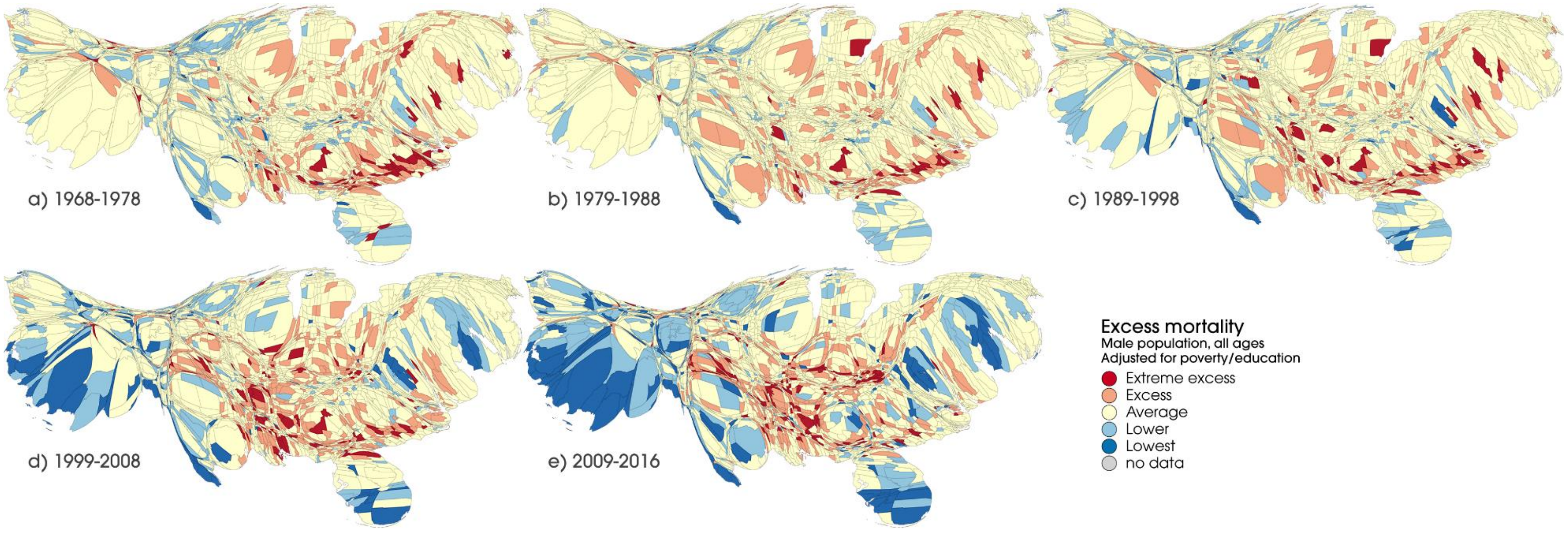

Fig. S5. Excess mortality by US county, adjusted for age, poverty and education, males, all ages, 1968/78 to 2009/16.

Source: National Center for Health Statistics (Compressed Mortality Files 1968-88, 1989-98, 1999-2016)

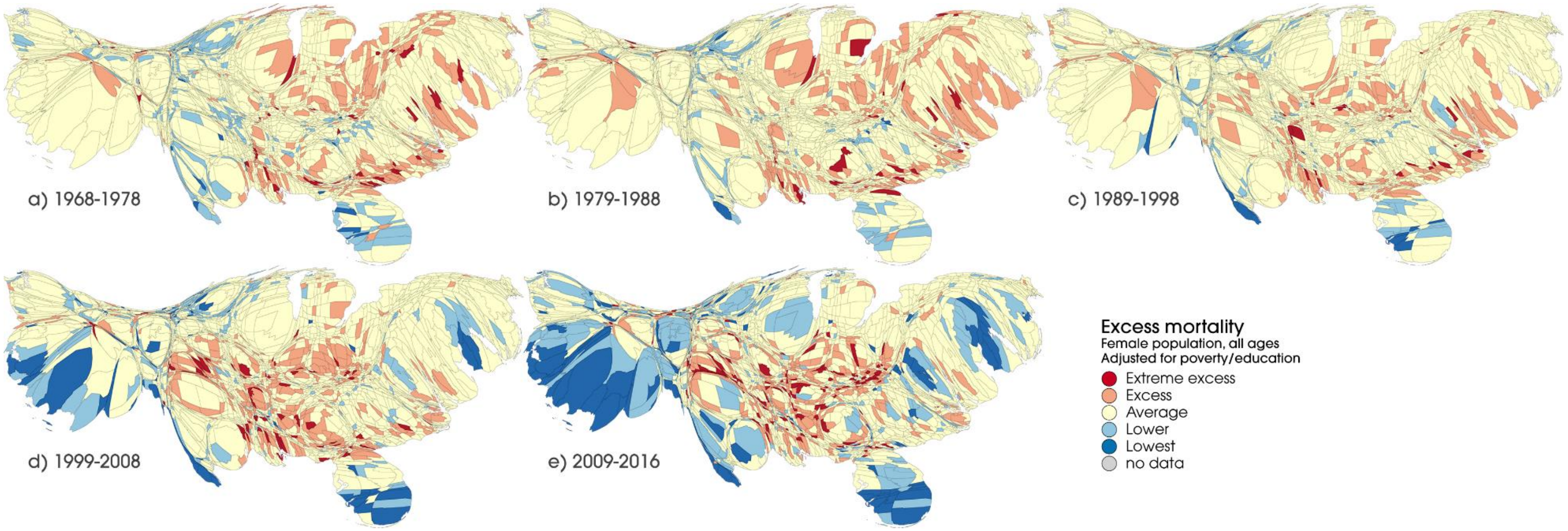

Fig. S6. Excess mortality by US county, adjusted for age, poverty and education, females, all ages, 1968/78 to 2009/16.

Source: National Center for Health Statistics (Compressed Mortality Files 1968-88, 1989-98, 1999-2016)

Table S4. Distribution of counties with excess mortality across US States by time period, males 0-64 years.

	Percentage of all counties with excess mortality in each period				
**State**	**1968-1978**	**1979-1988**	**1989-1998**	**1999-2008**	**2009-2016**
Alabama	4.9	3.4	4.3	3.8	4.4
Alaska	0.2	0.2	0.1	0.1	0.1
Arizona	1.4	1.3	1.0	0.5	0.4
Arkansas	0.6	1.2	2.9	4.5	4.7
California	1.5	4.2	3.3	0.6	0.6
Colorado	1.2	1.0	1.0	0.7	1.0
Connecticut	0.0	0.0	0.1	0.0	0.0
Delaware	0.3	0.2	0.4	0.2	0.1
District Of Columbia	0.2	0.2	0.1	0.1	0.1
Florida	5.4	4.7	6.0	3.7	2.1
Georgia	13.7	10.9	9.4	9.1	5.4
Hawaii	0.0	0.0	0.1	0.0	0.0
Idaho	0.9	0.8	0.3	0.4	0.5
Illinois	1.4	1.2	2.3	2.9	2.7
Indiana	2.2	2.0	1.7	4.8	4.6
Iowa	0.2	0.3	0.7	1.0	1.5
Kansas	1.4	2.2	2.4	4.5	4.9
Kentucky	2.2	1.5	1.0	2.7	5.7
Louisiana	3.1	3.5	3.4	3.1	2.9
Maine	0.2	0.2	0.0	0.0	0.0
Maryland	0.9	0.5	1.4	0.8	1.0
Massachusetts	0.6	0.5	0.4	0.0	0.0
Michigan	1.7	1.7	1.0	1.5	0.8
Minnesota	0.0	0.0	0.0	0.0	0.3
Mississippi	4.8	5.9	5.9	5.1	4.1
Missouri	1.4	1.3	1.9	3.8	4.4
Montana	3.4	2.7	1.3	1.4	1.3

Nebraska	0.8	1.0	1.0	1.2	1.5
Nevada	2.2	2.0	1.7	1.4	0.7
New Hampshire	0.3	0.3	0.0	0.0	0.0
New Jersey	0.6	1.2	1.6	0.6	0.5
New Mexico	1.7	1.3	0.9	0.5	0.8
New York	1.5	1.2	1.1	0.1	0.1
North Carolina	7.1	6.4	7.0	4.3	1.5
North Dakota	0.5	0.5	0.4	0.6	0.8
Ohio	2.5	2.0	1.4	3.4	2.3
Oklahoma	1.4	3.7	4.6	5.7	6.8
Oregon	0.9	1.2	1.1	0.9	0.5
Pennsylvania	1.9	2.0	1.7	1.3	1.9
Rhode Island	0.0	0.0	0.0	0.0	0.0
South Carolina	5.7	6.0	5.7	4.1	2.1
South Dakota	2.0	2.0	1.1	0.9	1.1
Tennessee	1.5	1.0	1.6	5.6	7.1
Texas	3.9	6.4	6.7	7.1	8.8
Utah	0.8	1.0	0.4	0.8	0.7
Vermont	0.3	0.0	0.1	0.0	0.0
Virginia	5.1	4.7	5.6	3.5	5.0
Washington	0.8	0.7	0.9	0.4	0.2
West Virginia	2.9	2.2	1.1	1.0	2.6
Wisconsin	0.3	0.2	0.4	0.9	0.3
Wyoming	1.9	1.7	1.1	0.7	0.7
Total	100.0	100.1	100.0	100.0	100.1

Values above 100% are due to rounding

Table S5. Distribution of counties with excess mortality across US States by time period, females 0-64 years.

	Percentage of all counties with excess mortality in each period				
**State**	**1968-1978**	**1979-1988**	**1989-1998**	**1999-2008**	**2009-2016**
Alabama	3.6	2.6	3.4	3.5	3.9
Alaska	0.1	0.2	0.2	0.1	0.1
Arizona	0.9	0.8	0.5	0.4	0.2
Arkansas	1.6	1.7	3.1	3.5	4.6
California	3.2	4.1	2.9	1.2	0.7
Colorado	1.3	0.0	0.0	0.9	1.1
Connecticut	0.0	0.0	0.0	0.0	0.0
Delaware	0.3	0.5	0.5	0.3	0.1
District Of Columbia	0.1	0.2	0.2	0.1	0.1
Florida	4.0	4.1	4.1	3.3	2.8
Georgia	13.0	10.9	12.7	8.5	5.7
Hawaii	0.0	0.0	0.0	0.0	0.0
Idaho	0.9	0.8	0.9	0.4	0.7
Illinois	1.7	2.5	1.7	3.1	3.4
Indiana	2.7	2.5	2.9	6.1	4.8
Iowa	0.1	0.0	0.3	1.0	1.5
Kansas	0.9	1.2	1.9	4.6	4.7
Kentucky	2.0	2.5	1.2	2.7	5.6
Louisiana	4.0	5.3	5.0	3.4	3.3
Maine	0.1	0.0	0.0	0.0	0.0
Maryland	1.6	1.5	1.5	0.7	0.6
Massachusetts	0.3	0.2	0.3	0.0	0.0
Michigan	3.3	2.3	1.5	2.1	0.7
Minnesota	0.0	0.0	0.2	0.1	0.2
Mississippi	4.3	5.6	6.4	4.8	3.9
Missouri	1.3	2.0	1.9	3.9	5.3
Montana	2.5	2.3	1.2	1.4	1.6

Nebraska	0.4	0.2	0.9	1.2	1.9
Nevada	2.2	2.0	1.7	1.4	1.0
New Hampshire	0.3	0.0	0.0	0.0	0.0
New Jersey	1.7	1.7	1.7	0.5	0.2
New Mexico	1.4	0.8	0.0	0.0	0.6
New York	3.2	2.5	1.9	0.0	0.0
North Carolina	5.6	5.0	5.2	3.0	1.2
North Dakota	0.6	0.3	0.7	0.5	1.0
Ohio	3.3	4.3	2.2	3.9	3.0
Oklahoma	0.4	2.8	4.8	7.6	7.0
Oregon	0.7	1.5	1.9	0.9	0.6
Pennsylvania	2.3	1.7	1.4	0.8	1.1
Rhode Island	0.0	0.0	0.0	0.0	0.0
South Carolina	5.9	5.6	5.5	2.5	1.4
South Dakota	1.4	1.0	1.4	0.9	1.0
Tennessee	1.0	1.3	1.9	5.2	7.1
Texas	4.8	4.6	6.2	7.8	7.9
Utah	0.4	0.5	0.5	0.7	0.9
Vermont	0.0	0.0	0.0	0.0	0.0
Virginia	4.8	5.0	5.2	2.7	4.1
Washington	1.2	1.3	0.3	0.5	0.2
West Virginia	2.2	3.1	0.9	1.7	2.9
Wisconsin	0.6	0.7	0.3	0.5	0.3
Wyoming	1.6	0.7	1.0	1.4	1.3
Total	100.0	100.1	100.0	100.0	100.0

Values above 100% are due to rounding

Table S6. Distribution of counties with excess mortality across US States by time period, males, all ages.

	Percentage of all counties with excess mortality in each period				
**State**	**1968-1978**	**1979-1988**	**1989-1998**	**1999-2008**	**2009-2016**
Alabama	4.1	2.5	3.4	4.3	5.2
Alaska	0.2	0.3	0.0	0.0	0.0
Arizona	0.4	0.0	0.0	0.0	0.0
Arkansas	0.2	0.8	3.2	3.1	4.7
California	1.1	1.4	0.5	0.0	0.1
Colorado	0.9	0.0	0.0	0.5	0.1
Connecticut	0.0	0.0	0.0	0.0	0.0
Delaware	0.4	0.0	0.3	0.0	0.0
District Of Columbia	0.2	0.3	0.3	0.2	0.0
Florida	1.1	1.7	1.6	1.2	0.6
Georgia	14.9	13.4	11.6	11.2	6.3
Hawaii	0.0	0.0	0.0	0.0	0.0
Idaho	0.4	0.3	0.3	0.0	0.6
Illinois	2.6	2.0	2.6	3.6	3.6
Indiana	3.5	3.1	3.7	5.6	6.0
Iowa	0.4	0.6	0.5	1.7	1.8
Kansas	1.3	1.1	2.6	3.8	4.6
Kentucky	2.2	2.5	2.6	5.1	8.2
Louisiana	3.5	4.5	5.5	4.1	3.3
Maine	1.1	0.0	0.5	0.0	0.0
Maryland	0.9	0.8	0.5	0.8	0.4
Massachusetts	0.7	0.3	0.5	0.0	0.0
Michigan	2.2	2.0	1.1	0.7	0.3
Minnesota	0.0	0.0	0.0	0.2	0.6
Mississippi	5.8	5.9	6.3	6.6	5.6
Missouri	2.2	1.7	1.8	3.0	3.5
Montana	2.6	2.0	2.1	1.2	1.0

Nebraska	0.2	1.4	1.1	1.2	1.1
Nevada	2.4	3.4	2.1	1.5	0.7
New Hampshire	0.2	0.3	0.3	0.0	0.0
New Jersey	0.7	1.1	0.8	0.3	0.4
New Mexico	0.4	0.6	0.0	0.0	0.1
New York	2.4	2.5	1.6	0.0	0.0
North Carolina	7.5	8.1	7.9	3.3	1.7
North Dakota	0.4	0.6	0.5	0.7	1.3
Ohio	3.7	4.8	2.9	5.6	3.2
Oklahoma	0.4	1.7	3.2	6.9	7.0
Oregon	0.7	0.3	0.5	0.2	0.1
Pennsylvania	3.9	3.4	2.4	1.8	2.4
Rhode Island	0.0	0.0	0.0	0.0	0.0
South Carolina	7.1	5.3	5.3	3.3	2.1
South Dakota	1.9	2.2	1.6	1.2	1.1
Tennessee	1.3	1.4	2.1	5.5	7.5
Texas	1.5	3.9	4.7	4.1	6.4
Utah	0.0	0.8	0.0	0.2	0.4
Vermont	0.4	0.3	0.0	0.0	0.0
Virginia	6.5	6.2	7.4	3.6	3.8
Washington	0.2	0.0	0.3	0.0	0.0
West Virginia	3.5	3.4	2.9	2.3	3.3
Wisconsin	0.7	0.6	0.8	0.7	0.6
Wyoming	1.5	1.1	0.3	0.8	0.4
Total	100.1	100.1	100.0	100.0	100.0

Values above 100% are due to rounding

Table S7. Distribution of counties with excess mortality across US States by time period, females, all ages.

	Percentage of all counties with excess mortality in each period				
**State**	**1968-1978**	**1979-1988**	**1989-1998**	**1999-2008**	**2009-2016**
Alabama	2.4	1.4	2.4	3.8	4.9
Alaska	0.2	0.3	0.0	0.0	0.0
Arizona	0.2	0.3	0.0	0.0	0.0
Arkansas	0.0	1.1	2.4	3.0	4.1
California	0.7	2.6	2.1	0.6	0.0
Colorado	0.2	0.3	0.0	0.4	0.2
Connecticut	0.0	0.0	0.0	0.0	0.0
Delaware	0.2	0.3	0.6	0.0	0.0
District Of Columbia	0.2	0.3	0.3	0.0	0.0
Florida	1.2	1.1	3.0	2.1	1.1
Georgia	9.5	7.9	10.1	10.4	6.1
Hawaii	0.0	0.0	0.0	0.0	0.0
Idaho	0.5	0.3	0.9	0.4	0.8
Illinois	2.1	1.7	1.5	2.8	3.3
Indiana	5.0	4.3	5.1	5.1	5.6
Iowa	0.2	0.0	0.0	0.0	1.2
Kansas	0.5	0.9	1.2	2.6	3.6
Kentucky	4.3	4.3	6.3	6.8	9.9
Louisiana	5.4	8.5	7.1	4.3	3.3
Maine	1.0	0.3	0.0	0.0	0.2
Maryland	1.0	2.0	2.4	0.8	0.8
Massachusetts	0.5	0.6	0.3	0.0	0.0
Michigan	4.7	2.6	1.8	1.3	0.8
Minnesota	0.2	0.0	0.3	0.0	0.5
Mississippi	6.9	4.8	3.6	5.7	4.3
Missouri	1.7	0.9	3.0	3.6	3.8
Montana	1.2	2.0	1.5	1.5	1.8

Nebraska	0.5	0.0	0.3	0.6	1.2
Nevada	2.1	2.8	1.8	1.7	0.9
New Hampshire	0.2	0.0	0.0	0.0	0.0
New Jersey	2.1	2.8	2.1	0.4	0.2
New Mexico	0.5	0.0	0.0	0.0	0.2
New York	5.2	6.2	1.5	0.2	0.2
North Carolina	4.3	2.6	4.8	3.2	1.4
North Dakota	0.5	0.3	0.3	0.4	0.9
Ohio	7.6	6.5	5.7	5.8	3.5
Oklahoma	0.5	1.4	2.4	8.3	7.1
Oregon	0.0	0.6	0.6	0.4	0.0
Pennsylvania	6.4	5.4	1.5	0.8	1.2
Rhode Island	0.0	0.0	0.0	0.0	0.0
South Carolina	6.4	4.8	3.3	1.3	0.6
South Dakota	1.2	1.1	0.6	0.8	0.9
Tennessee	1.0	1.1	1.8	4.3	7.4
Texas	1.4	1.7	3.9	6.8	7.1
Utah	0.2	0.3	0.3	0.8	1.2
Vermont	0.0	0.3	0.3	0.2	0.0
Virginia	4.0	7.4	7.4	3.8	4.3
Washington	0.0	0.3	0.3	0.2	0.0
West Virginia	4.3	4.8	4.5	3.6	4.6
Wisconsin	1.0	0.6	0.3	0.4	0.2
Wyoming	1.0	0.6	0.9	1.3	1.1
Total	100.1	100.0	100.0	100.0	100.0

Values above 100% are due to rounding

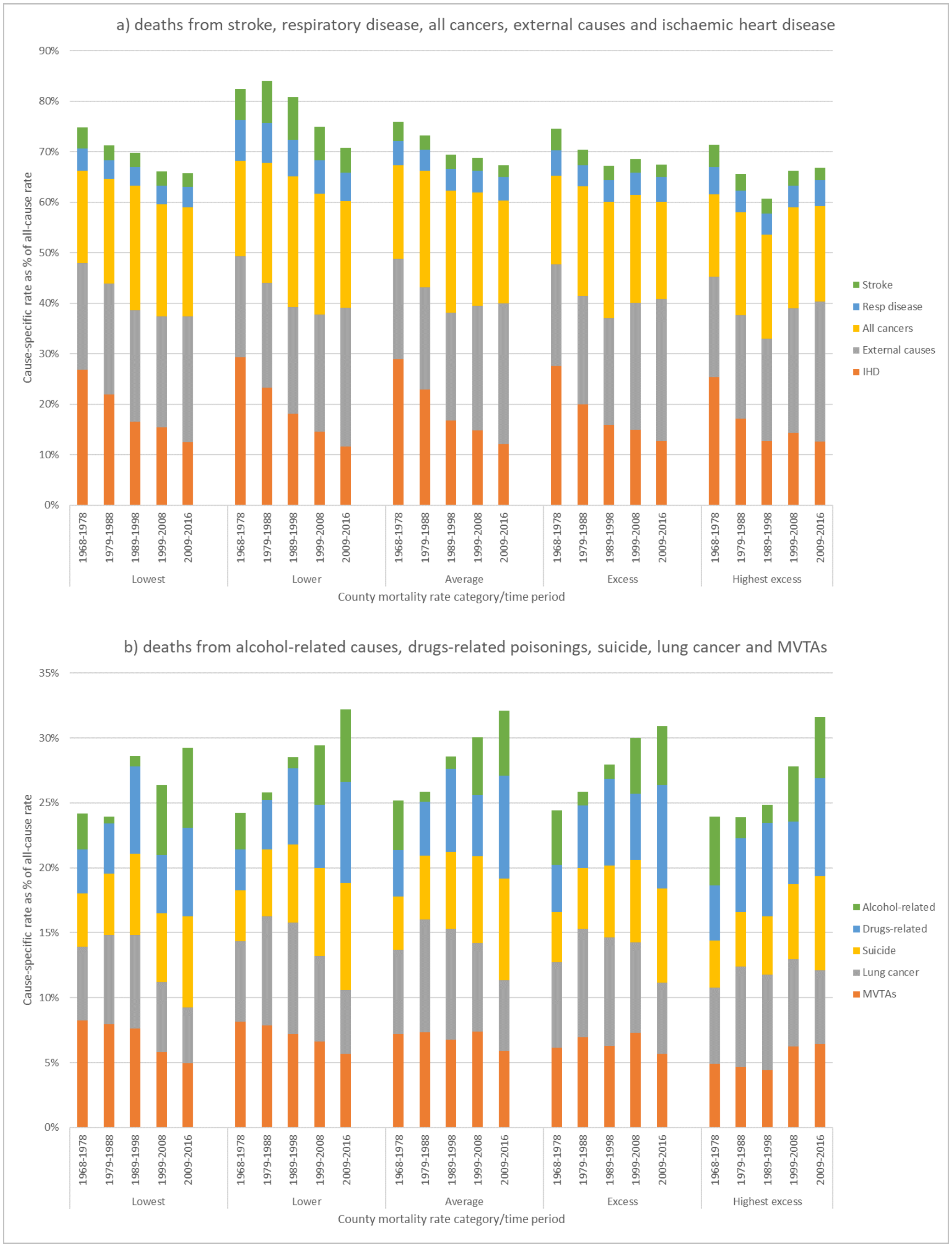

Fig. S7. Contribution of different causes of death in each time period by counties grouped by levels of adjusted mortality: males, 0-64 years. Cause-specific mortality rates are shown as a percentage of all-cause mortality rates. Note different axis scales in chart a and b. *Source: National Center for Health Statistics (Compressed Mortality Files 1968-88, 1989-98, 1999-2016)*

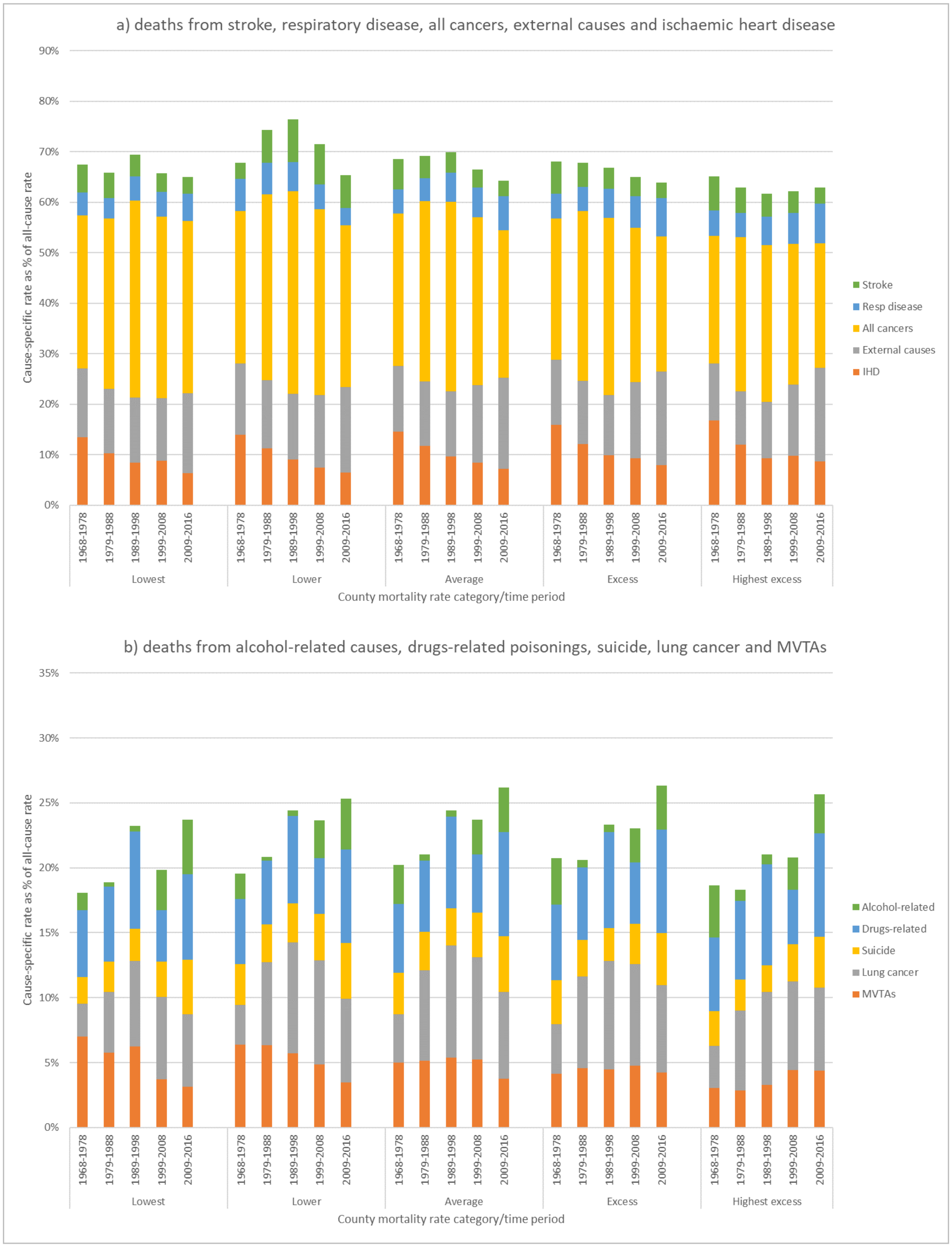

Fig. S8. Contribution of different causes of death in each time period by counties grouped by levels of adjusted mortality: females, 0-64 years. Cause-specific mortality rates are shown as a percentage of all-cause mortality rates. Note different axis scales in chart a and b. *Source: National Center for Health Statistics (Compressed Mortality Files 1968-88, 1989-98, 1999-2016)*

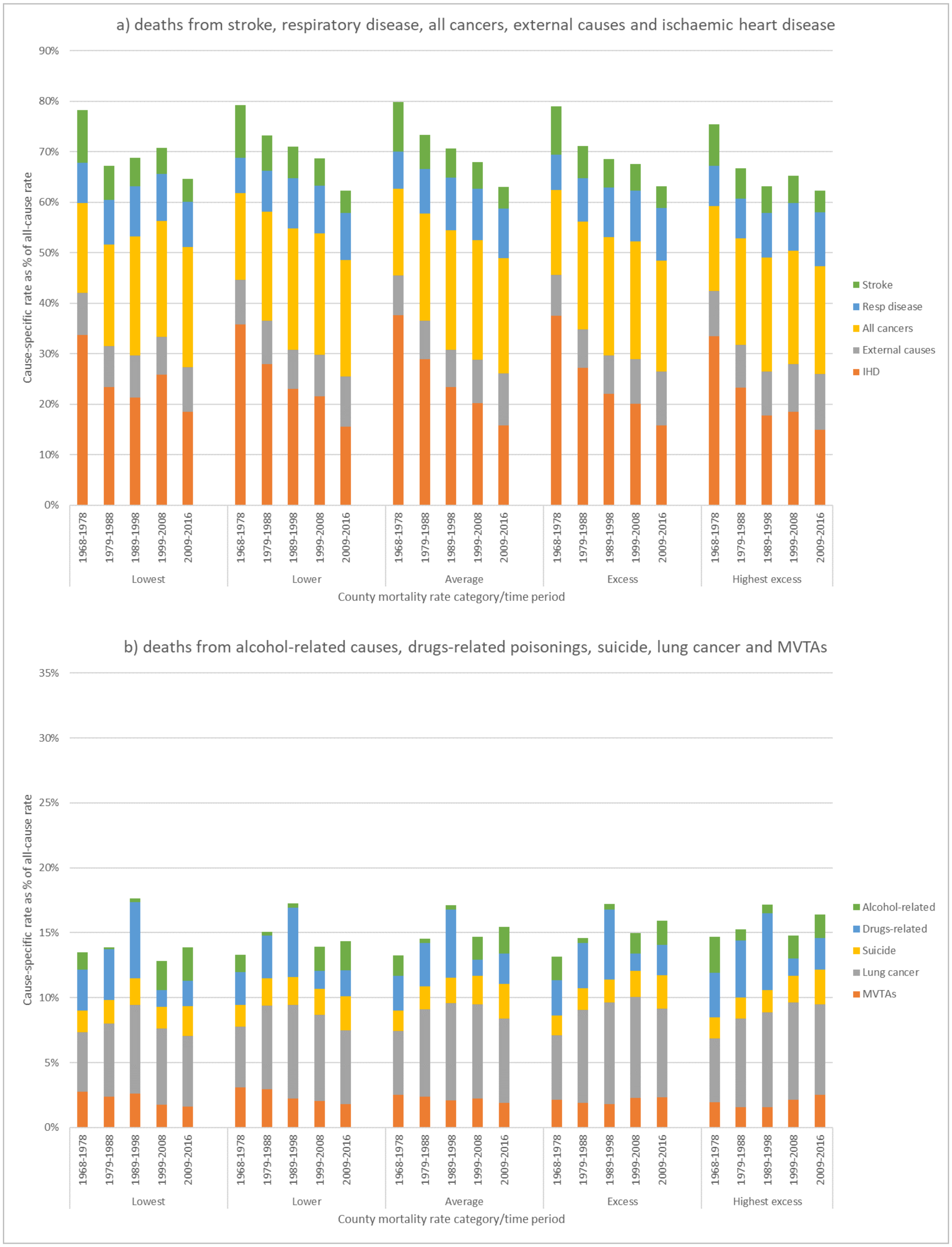

Fig. S9. Contribution of different causes of death in each time period by counties grouped by levels of adjusted mortality: males, all ages. Cause-specific mortality rates are shown as a percentage of all-cause mortality rates. Note different axis scales in chart a and b. *Source: National Center for Health Statistics (Compressed Mortality Files 1968-88, 1989-98, 1999-2016)*

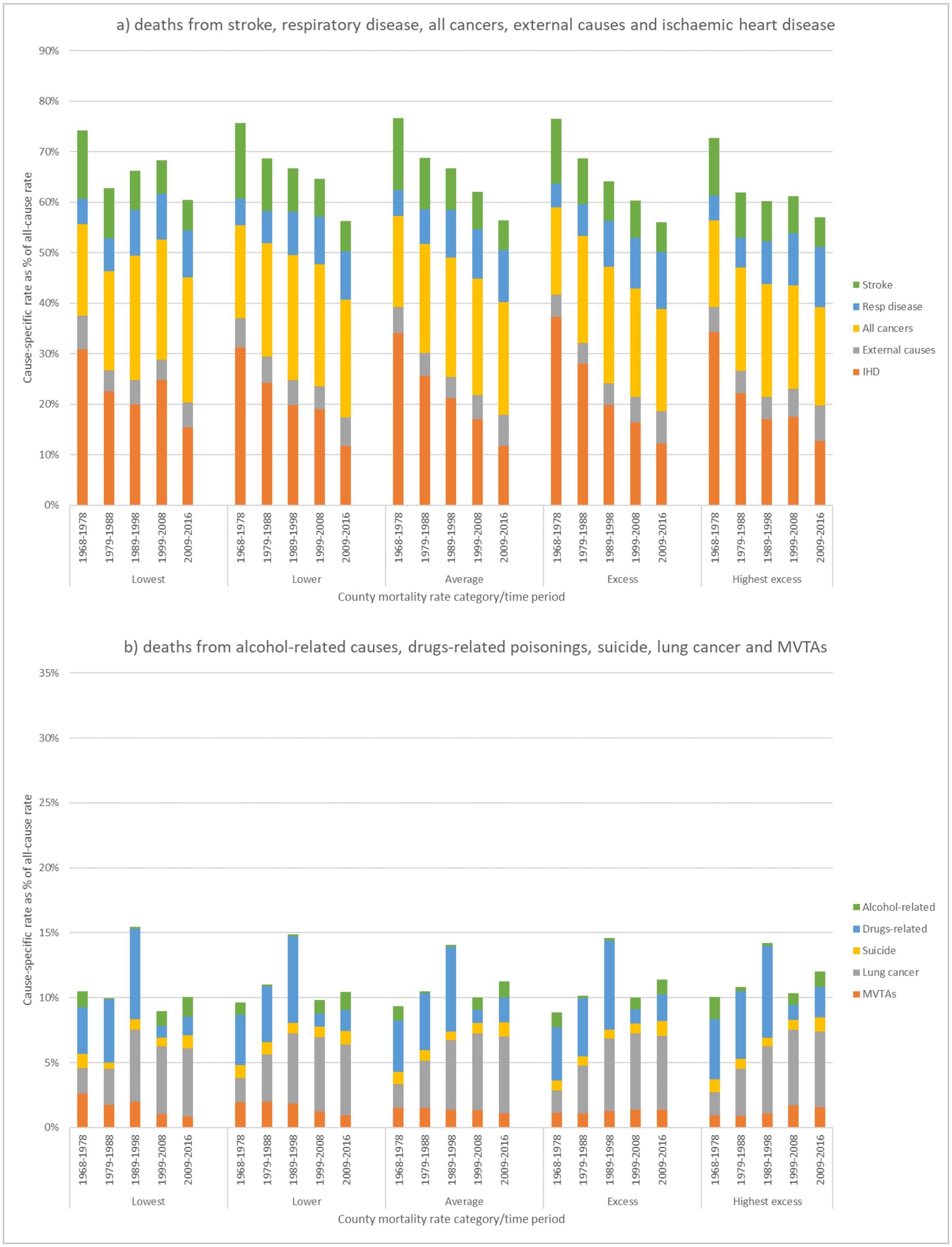

Fig. S10. Contribution of different causes of death in each time period by counties grouped by levels of adjusted mortality: females, all ages. Cause-specific mortality rates are shown as a percentage of all-cause mortality rates. Note different axis scales in chart a and b. *Source: National Center for Health Statistics (Compressed Mortality Files 1968-88, 1989-98, 1999-2016)*

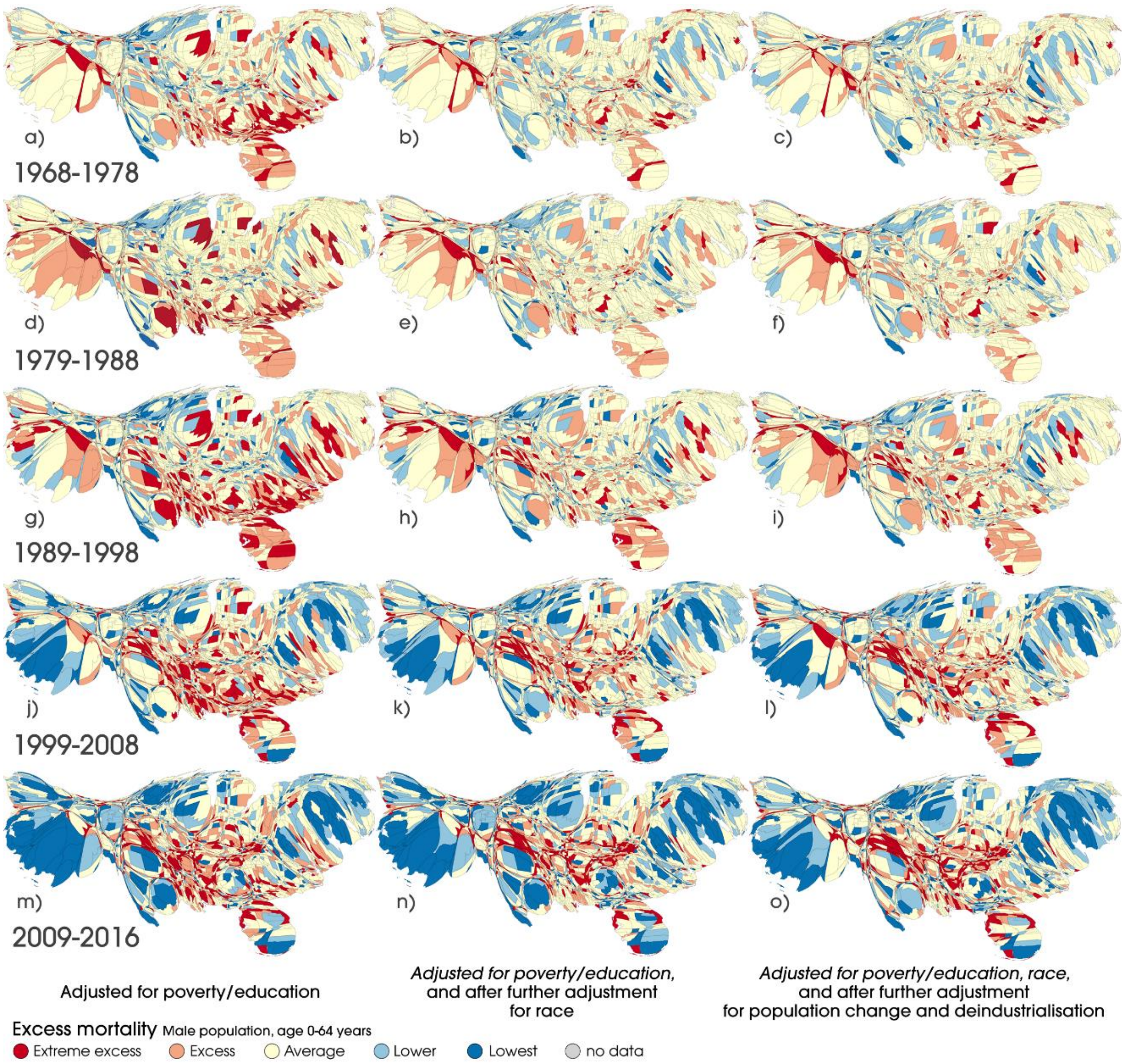

Fig. S11. Excess mortality by US county, adjusted for (1) age, poverty and education, (2) age, poverty, education and race and (3) age, poverty, education, race, population change and deindustrialisation, <u>males, 0-64 years</u>.

Source: National Center for Health Statistics (Compressed Mortality Files 1968-88, 1989-98, 1999-2016)

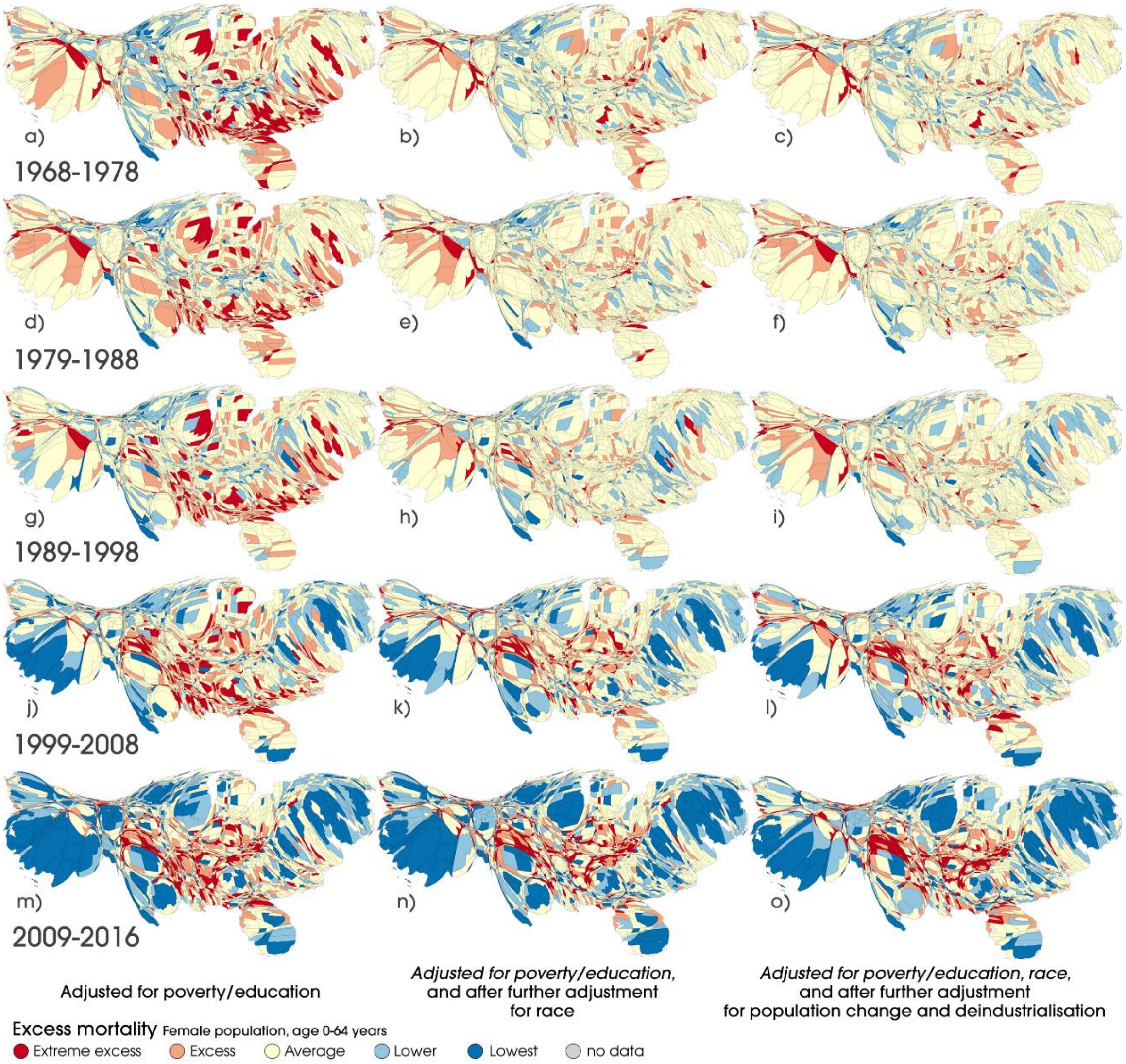

Fig. S12. Excess mortality by US county, adjusted for (1) age, poverty and education, (2) age, poverty, education and race and (3) age, poverty, education, race, population change and deindustrialisation, females, 0-64 years.

Source: National Center for Health Statistics (Compressed Mortality Files 1968-88, 1989-98, 1999-2016)

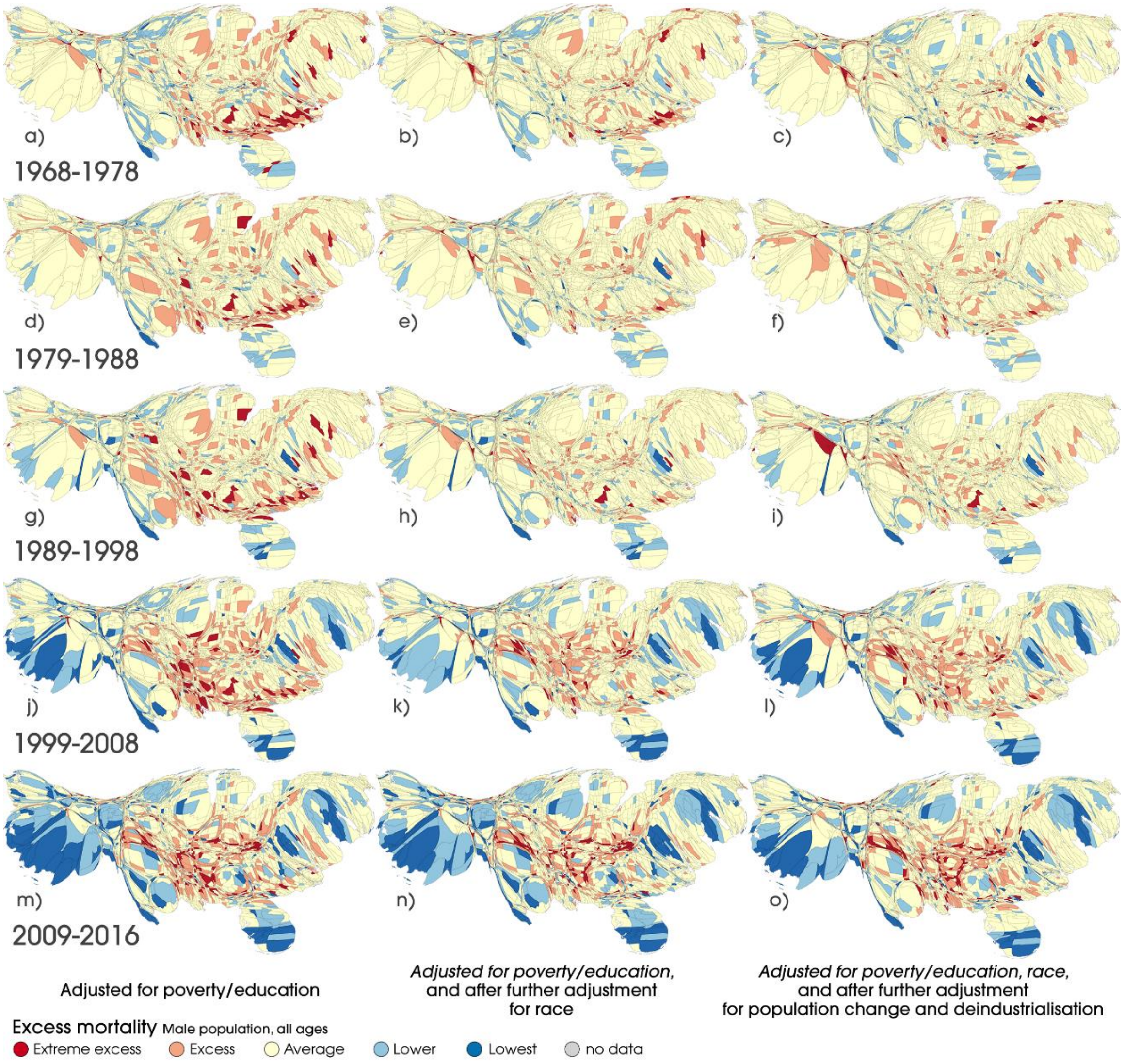

Fig. S13. Excess mortality by US county, adjusted for (1) age, poverty and education, (2) age, poverty, education and race and (3) age, poverty, education, race, population change and deindustrialisation, males, all ages.

Source: National Center for Health Statistics (Compressed Mortality Files 1968-88, 1989-98, 1999-2016)

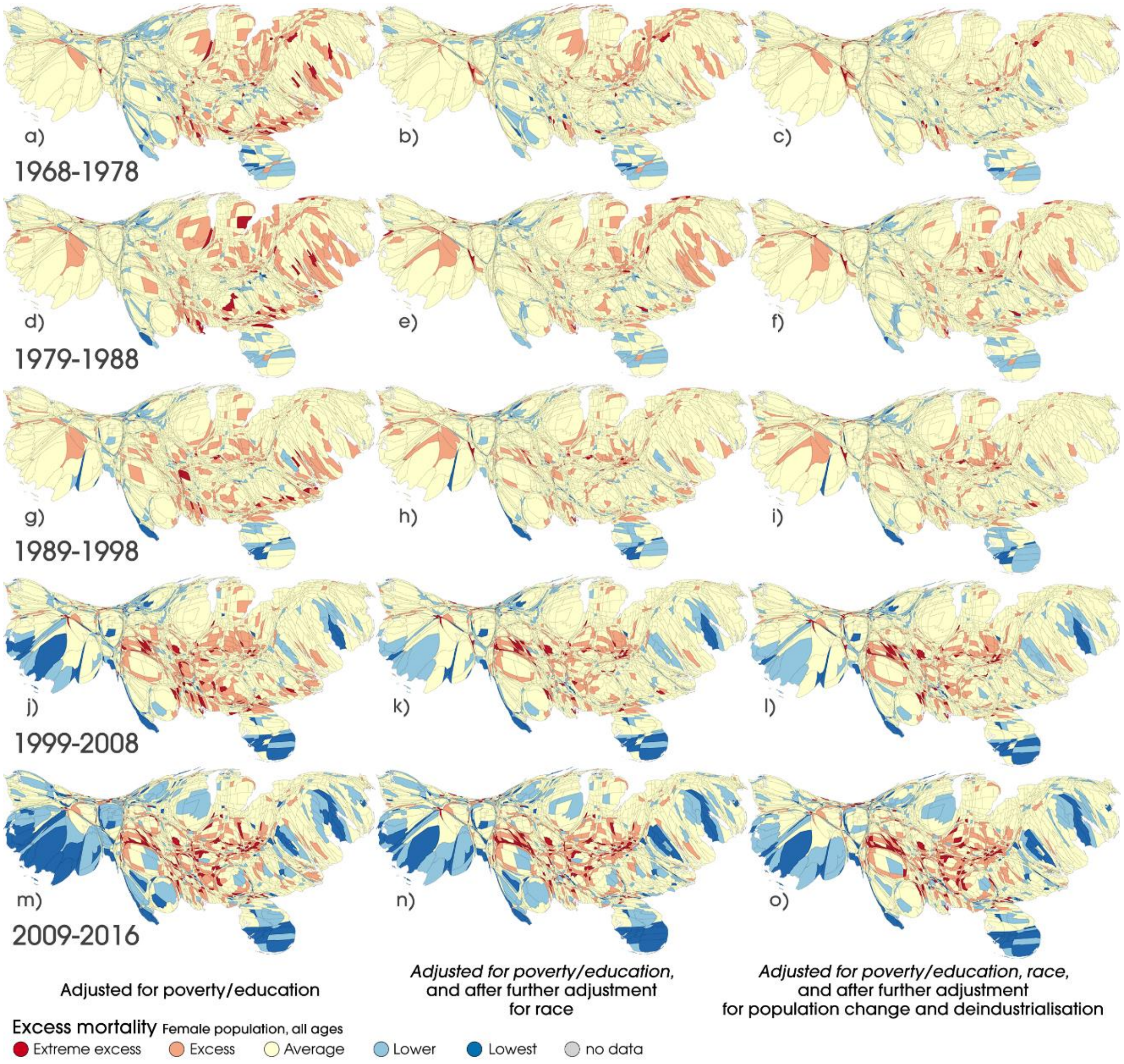

Fig. S14. Excess mortality by US county, adjusted for (1) age, poverty and education, (2) age, poverty, education and race and (3) age, poverty, education, race, population change and deindustrialisation, females all ages.

Source: National Center for Health Statistics (Compressed Mortality Files 1968-88, 1989-98, 1999-201

Table S8: Proportion of counties in very low, low, average, high and very high excess mortality groups for each model over different time periods.

(a) Males: all ages and 0-64 years

		**Males: all ages**			**Males: 0-64 years**		
**Time Period**	**Excess Level**	**Model 1**	**Model 2**	**Model 3**	**Model 1**	**Model 2**	**Model 3**
**1968-1978**	Lowest (>20% below average)	0.9	0.5	0.5	3.8	2.0	1.9
	Low (20-10% below average)	12.1	9.4	7.8	16.0	12.8	12.4
	Average	71.1	77.2	80.6	58.4	67.6	68.5
	High (10-20% excess)	13.0	11.0	8.5	13.7	12.1	12.0
	Highest (>20% excess)	2.9	1.9	2.6	8.2	5.6	5.3
**1979-1988**	Lowest (>20% below average)	0.7	0.4	0.3	3.5	2.0	1.8
	Low (20-10% below average)	8.8	7.6	7.3	13.7	12.6	12.6
	Average	78.2	81.9	82.8	62.8	69.2	69.5
	High (10-20% excess)	10.6	7.9	7.6	13.7	11.7	11.6
	Highest (>20% excess)	1.7	2.1	2.0	6.4	4.5	4.5
**1989-1998**	Lowest (>20% below average)	1.5	1.0	0.9	5.3	3.9	3.6
	Low (20-10% below average)	9.3	8.6	8.0	14.5	12.9	13.5
	Average	77.0	79.1	81.1	56.8	63.1	62.7

	High (10-20% excess)	9.9	10.1	8.9	14.1	14.3	14.2
	Highest (>20% excess)	2.4	1.1	1.2	9.3	5.8	6.0
**1999-2008**	Lowest (>20% below average)	4.6	3.5	3.5	8.6	8.6	8.3
	Low (20-10% below average)	11.4	11.5	11.2	14.4	14.3	15.0
	Average	64.7	67.7	67.3	48.5	49.7	49.8
	High (10-20% excess)	15.6	14.4	15.2	18.0	17.3	15.9
	Highest (>20% excess)	3.8	3.0	2.8	10.4	10.1	11.0
**2009-2016**	Lowest (>20% below average)	6.0	5.9	5.1	10.6	11.8	11.3
	Low (20-10% below average)	12.2	12.2	11.9	13.9	13.5	14.1
	Average	58.7	58.6	60.9	44.2	44.0	44.5
	High (10-20% excess)	17.0	17.1	16.0	16.2	14.7	15.0
	Highest (>20% excess)	6.0	6.1	6.0	15.1	15.9	15.2

Model 1: adjusted for age + poverty + education; model 2: adjusted for age + poverty + education + race; model 3: adjusted for age + poverty + education + race + deindustrialization + population change

(b) Females: all ages and 0-64 years

		**Females: all ages**			**Females: 0-64 years**		
**Time Period**	**Excess Level**	**Model 1**	**Model 2**	**Model 3**	**Model 1**	**Model 2**	**Model 3**
**1968-1978**	Lowest (>20% below average)	0.6	0.4	0.3	4.1	0.7	0.5
	Low (20-10% below average)	10.4	8.3	6.0	17.2	12.4	11.9
	Average	74.6	79.8	84.8	55.4	71.2	73.0
	High (10-20% excess)	12.9	9.5	6.9	13.7	11.6	10.6
	Highest (>20% excess)	1.6	2.0	2.0	9.6	4.2	4.0
**1979-1988**	Lowest (>20% below average)	0.5	0.2	0.1	2.6	0.6	0.6
	Low (20-10% below average)	8.8	7.5	7.4	15.0	10.1	9.5
	Average	78.5	82.1	83.3	62.2	76.0	77.6
	High (10-20% excess)	10.9	9.1	7.3	15.0	9.8	9.0
	Highest (>20% excess)	1.3	1.0	1.8	5.3	3.4	3.3
**1989-1998**	Lowest (>20% below average)	1.0	0.7	0.7	2.9	1.3	1.0
	Low (20-10% below average)	8.7	8.0	8.3	13.3	11.2	10.8
	Average	79.5	80.9	80.7	64.3	73.6	74.3
	High (10-20% excess)	9.7	9.5	9.4	14.1	11.6	11.8
	Highest (>20% excess)	1.0	0.9	0.9	5.4	2.3	2.0

**1999-2008**	Lowest (>20% below average)	3.3	2.2	2.2	5.9	5.9	5.5
	Low (20-10% below average)	10.7	10.2	10.3	14.6	14.6	15.3
	Average	69.1	72.7	72.5	54.1	55.3	55.1
	High (10-20% excess)	14.4	12.4	12.3	16.3	15.0	16.0
	Highest (>20% excess)	2.6	2.5	2.7	9.1	9.3	8.1
**2009-2016**	Lowest (>20% below average)	5.3	4.4	4.0	10.6	11.8	10.9
	Low (20-10% below average)	12.7	12.4	12.8	13.9	14.4	15.4
	Average	61.0	61.9	62.2	44.5	43.1	44.3
	High (10-20% excess)	15.6	16.2	16.0	17.3	14.6	14.0
	Highest (>20% excess)	5.5	5.2	5.0	13.7	16.0	15.4

Model 1: adjusted for age + poverty + education; model 2: adjusted for age + poverty + education + race; model 3: adjusted for age + poverty + education + race + deindustrialization + population change
